# Structural and Functional Analyses of *Trypanosoma brucei* Nucleoside Diphosphate Kinase

**DOI:** 10.1021/acsomega.5c11614

**Published:** 2026-03-09

**Authors:** Patricia Makori, Michael P. Boeckman, Heidi S. David, Finley Payne, Markenya Gatling, Colby Greer, Dylan Hayes, Alexandra Jefferson, Micaela Maxwell, Christian Smith, Jamilah Watson, London Williams, Jazmin Barkley, Caitlyn Pepper, Tawanda Zininga, Sandhya Subramanian, Ariel Abramov, Anna S. Gardberg, Thomas E. Edwards, Bart L. Staker, Lance J. Stewart, Peter J. Myler, Oluwatoyin A Asojo, Olamide Jeje, Sylvia Fanucchi, Ikechukwu Achilonu, Craig L. Smith, Graham Chakafana

**Affiliations:** † Department of Chemistry and Biochemistry, 3726 Hampton University, Hampton, Virginia 23668, United States; ‡ Department of Biology, 7548 Washington University in St. Louis, St. Louis, Missouri 63110, United States; § The Governor’s School, Hampton, Virginia 23666, United States; ∥ Department of Biochemistry, 26697 Stellenbosch University, Stellenbosch 7602, South Africa; ⊥ Center for Global Infectious Disease Research, Seattle Children’s Research Institute, 307 Westlake Avenue North Suite 500, Seattle, Washington 98109, United States; # Seattle Structural Genomics Center for Infectious Disease (SSGCID), Seattle, Washington 98105, United States; ∇ Beryllium Discovery Corp., Bainbridge Island, Washington 98110, United States; ○ Dartmouth Cancer Center & Department of Biochemistry and Cell Biology, Dartmouth Giesel School of Medicine, Lebanon, New Hampshire 03756, United States; ◆ Protein Structure−Function Research Laboratory, School of Molecular and Cell Biology, 37707 University of the Witwatersrand, Braamfontein, Johannesburg 2050, South Africa

## Abstract

*Trypanosoma brucei*, the causative agent of Human African Trypanosomiasis (HAT), relies exclusively on purine salvage for nucleotide biosynthesis, making its nucleotide-processing enzymes attractive drug targets. Here, we present a comprehensive structural and functional characterization of *T. brucei*’s nucleoside diphosphate kinase B (*Tb*NDPK), a key enzyme in nucleotide homeostasis. Circular dichroism and fluorescence spectroscopy revealed that *Tb*NDPK is highly stable under thermal and chemical stress and undergoes nucleotide-induced conformational changes. This study also presents high-resolution crystal structures of the *apo* enzyme and complexes with UDP, CDP, and GDP, showing a conserved hexameric fold, with induced-fit binding via a flexible loop involving Phe59 and key active-site residues. Enzymatic assays revealed substrate preferences for UDP and GDP, while deoxyribonucleotide diphosphates were processed with significantly reduced efficiency. Molecular dynamics simulations revealed ligand-dependent flexibility and subunit-specific nucleotide dynamics, indicating potential asymmetry and cooperative communication within the hexamer. Collectively, these findings position *Tb*NDPK as a thermostable, catalytically efficient, and structurally distinct enzyme optimized for ribonucleotide metabolism and support its potential as a selective target for future antitrypanosomal drug discovery.

## Graphical abstract

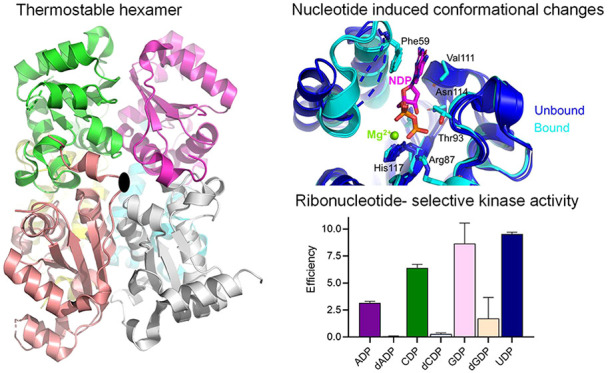

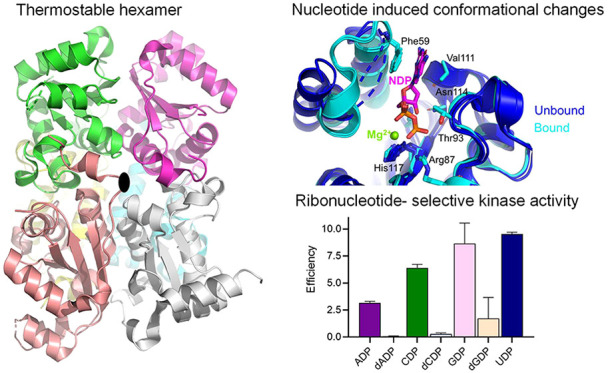

## Introduction

*Trypanosoma brucei* is the causative agent of Human African Trypanosomiasis (HAT) in humans and nagana in livestock. Both diseases pose significant health and economic threats.^1^ While *T. brucei* gambiense and *T. brucei* rhodesiense are associated with HAT, *T. brucei* brucei causes nagana. The close genetic and biological similarities of *T. brucei* subspecies present opportunities for developing broad-spectrum antitrypanosomal drugs that could effectively treat both diseases.^2^ With no significant advancements in treatment over the past five decades, HAT remains a Neglected Tropical Disease (NTD), underscoring the urgent need for new therapeutic options.^3–5^ While recent efforts have explored the potential development of new HAT vaccines, there are currently no approved vaccines to prevent all forms of trypanosomiasis.^6,7^ This emphasizes the need for effective drug treatments and vector control strategies. Although the overall number of HAT cases in endemic regions has decreased to less than 2000 over the past decades, the constant occurrence of HAT infections in nonendemic regions is concerning.^8,9^ The identification of new *T. brucei* drugs is therefore important. Nucleoside diphosphate kinases (NDPKs) are attractive targets for developing antimicrobials and antiparasitic agents due to their diverse biological functions and involvement in fundamental cellular functions, which include proliferation, differentiation, and transcription.^10,11^

NDPKs facilitate the transfer of a phosphoryl group from a nucleoside triphosphate to a nucleoside diphosphate through a ping-pong mechanism involving a phospho-histidine intermediate state.^12^ NDPKs are vital for maintaining intracellular nucleotide pools.^13^ Additionally, NDPKs are involved in phosphorylating other proteins that regulate various cellular functions.^14,15^ Beyond intracellular nucleotide homeostasis, NDPKs contribute to DNA damage responses, signal transduction, and virulence.^16–18^ Secreted NDPKs alter host cell signaling pathways,^18^ suggesting applications for *T. brucei* NDPK (*Tb*NDPK) in novel vaccine design.

NDPKs facilitate the conversion of free purines and pyrimidines into nucleosides and, subsequently, into nucleotides. The availability of pyrimidine-based nucleotides in cells depends on the activity of NDPKs.^19^ Unlike humans, which rely on both salvage and biosynthetic pathways for their purine requirements, *T. brucei* relies solely on purine salvage as it cannot synthesize purines de novo.^20^ Targeting a salvage pathway-regulating enzyme like *Tb*NDPK is an Achilles’ heel of *T. brucei* parasites since it regulates purine biosynthesis, essential for nucleotide pool maintenance.^13^ NDPK inhibition has been explored in other trypanosomatids including *Leishmania major* NDPKb, with inhibitors like BTB13319 and SU11652 (a sunitinib analog) emerging as promising scaffolds for rational drug design.^11,21^ Despite well-documented processes in other species, how *Tb*NDPK modulates different NDP-/dNDP-based substrates to maintain balanced NTP levels in the cell remains unclear. While NTP synthesis pathways in related trypanosomatids like *T. cruzi* and *Leishmania major* are known, insights into the protein machinery regulating nucleotide metabolism in *T. brucei* are lacking, with crucial experiments characterizing *Tb*NDPK still outstanding.

Given its potential as a drug target, understanding *Tb*NDPK’s structural and functional features is crucial for drug development. The roles of NDPKs in activating nucleoside analogs to their active triphosphate forms have previously been explored as an inhibition strategy.^22^ Despite the significance of *Tb*NDPK in nucleotide metabolism, the precise details of how it binds to and hydrolyzes its nucleotide substrates remain elusive. Further elucidation of these molecular interactions is essential for a comprehensive understanding of *Tb*NDPK’s functional mechanisms. This study elucidates *Tb*NDPK’s substrate binding and hydrolysis, providing critical insights that can inform drug development strategies and therapeutic interventions against trypanosomatid parasites. Kinase activity assays were conducted to delineate the substrate specificity of recombinant *Tb*NDPK, marking the first exploration of its enzymatic activity. The crystal structure of *Tb*NDPK is also reported, providing insights into the molecular basis of its distinctive substrate specificity. Overall, this study clarifies the structural and functional features of *Tb*NDPK, positioning it as a promising candidate for future drug development efforts.

## Results

### *Tb*NDPK Exhibits High Structural Stability

Recombinant *T. brucei* NDPK was successfully expressed and purified (Figure S1) and subjected to further analyses. CD spectroscopic analyses of recombinant *Tb*NDPK revealed a characteristic pattern of secondary structure, with a positive peak around 195 nm and two negative troughs at approximately 208 and 222 nm (Figure 1A), indicative of a predominantly α-helical structure. To evaluate the structural stability of *Tb*NDPK, we conducted thermal denaturation experiments. *Tb*NDPK exhibited high thermal stability, retaining 50% of its folded structure at temperatures exceeding 65 °C (Figure 1B–D). Such a high level of thermal resilience is noteworthy given the complex life cycle of *T. brucei*, which alternates between a homeothermic human host and a poikilothermic insect vector. The ability of *Tb*NDPK to maintain its structural integrity across a broad temperature range suggests a role for this protein in ensuring the parasite’s survival under diverse physiological conditions. Previous studies have shown that NDPK thermostability varies considerably across organisms and oligomeric states, and is influenced by multiple structural features.^23,24^ Moreover, in the presence of various nucleotides, we observed an improved resilience to thermal denaturation (Figure 1C,D). These findings highlight *Tb*NDPK’s resilience to thermal stress, suggesting that *Tb*NDPK is well-adapted to the varying physiological environments encountered during *T. brucei*’s life cycle.

**Figure 1:**
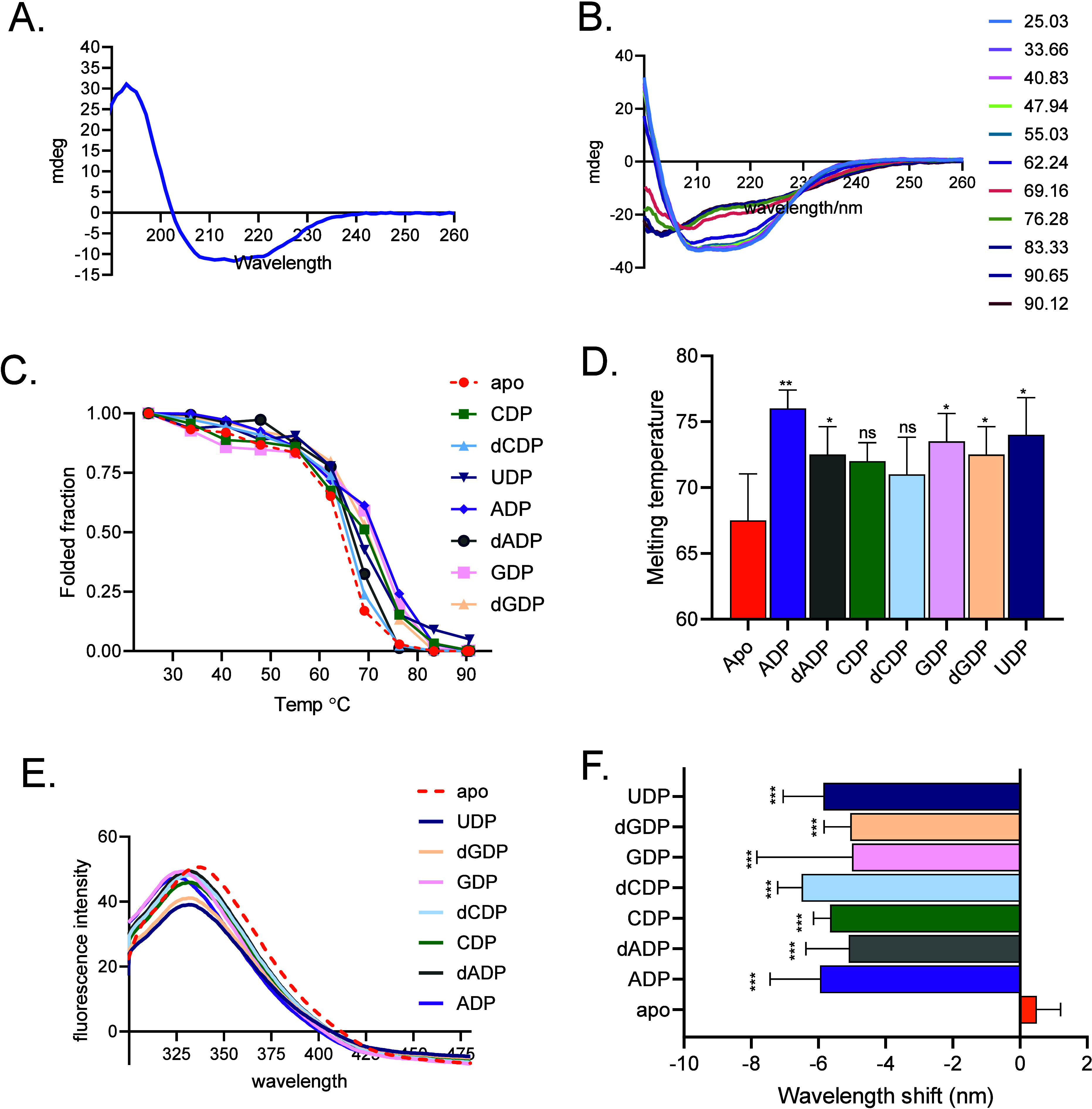
Biophysical analyses of *Tb*NDPK. (A) CD spectrum of *Tb*NDPK and (B) thermal denaturation analysis of *Tb*NDPK. (C, D) Thermal denaturation analyses of *Tb*NDPK in the presence of different nucleotides. (E) Intrinsic fluorescence emission spectra of *Tb*NDPK in the absence and presence of nucleotides. (F) Summary of fluorescence emission wavelength shifts upon nucleotide binding to TbNDPK. Data represent the mean of three independent replicates. Error bars indicate the standard deviation. Statistical significance is denoted by asterisks (*p < 0.05; **p < 0.01; ***p < 0.001) after one way ANOVA.

Given the biochemical role of NDPKs in nucleotide metabolism, we also examined the conformational dynamics of *Tb*NDPK upon nucleotide binding using tryptophan fluorescence spectroscopy. *Tb*NDPK possesses three tryptophan residues (W77, W132 and W141) located in the catalytic and oligomerization domains of the protein (Figure S2). Exposure to NDPs resulted in blue shifts in the fluorescence emission spectrum, indicating conformational changes in *Tb*NDPK relative to the unbound (*apo*) state (Figure 1E,F). These shifts suggest that *Tb*NDPK adopts an open conformation in its unbound state, transitioning to a closed conformation upon nucleotide binding. This conformational flexibility may be integral to its biochemical function, highlighting the dynamic nature of *Tb*NDPK in response to nucleotides.

### Structural Analyses Reveal That *Tb*NDPK Adopts a Prototypical NDPK Fold

The crystal structure of *T. brucei* NDPK (PDB ID: 4F36) was determined at 2.3 Å resolution and reveals a D3 hexameric enzyme with the canonical NDPK fold (Figure 2A,B). Each monomer adopts the characteristic α/β-sandwich architecture composed of a central four-stranded antiparallel β-sheet surrounded by helices, a fold that is highly conserved from bacteria to humans (Figure 2C). Superposition with previously determined structures (data not shown) demonstrates close alignment (RMSD values of <∼1 Å), highlighting the evolutionary robustness of the NDPK fold across diverse organisms. Central to the catalytic activity of NDPKs is the conserved HGS motif, which in our structure are residues His117, Gly118, and Ser119. In 4F36, His117 occupies a solvent-exposed position at the bottom of the active site where it undergoes transient phosphorylation, while Ser119 provides stabilization for the phosphohistidine intermediate, with Gly118 creating steric space for this interaction. The conservation of this motif strongly supports a shared catalytic mechanism in which a donor nucleoside triphosphate transfers its γ-phosphate to the active-site histidine before being relayed to an acceptor nucleoside diphosphate.^12,14^

**Figure 2:**
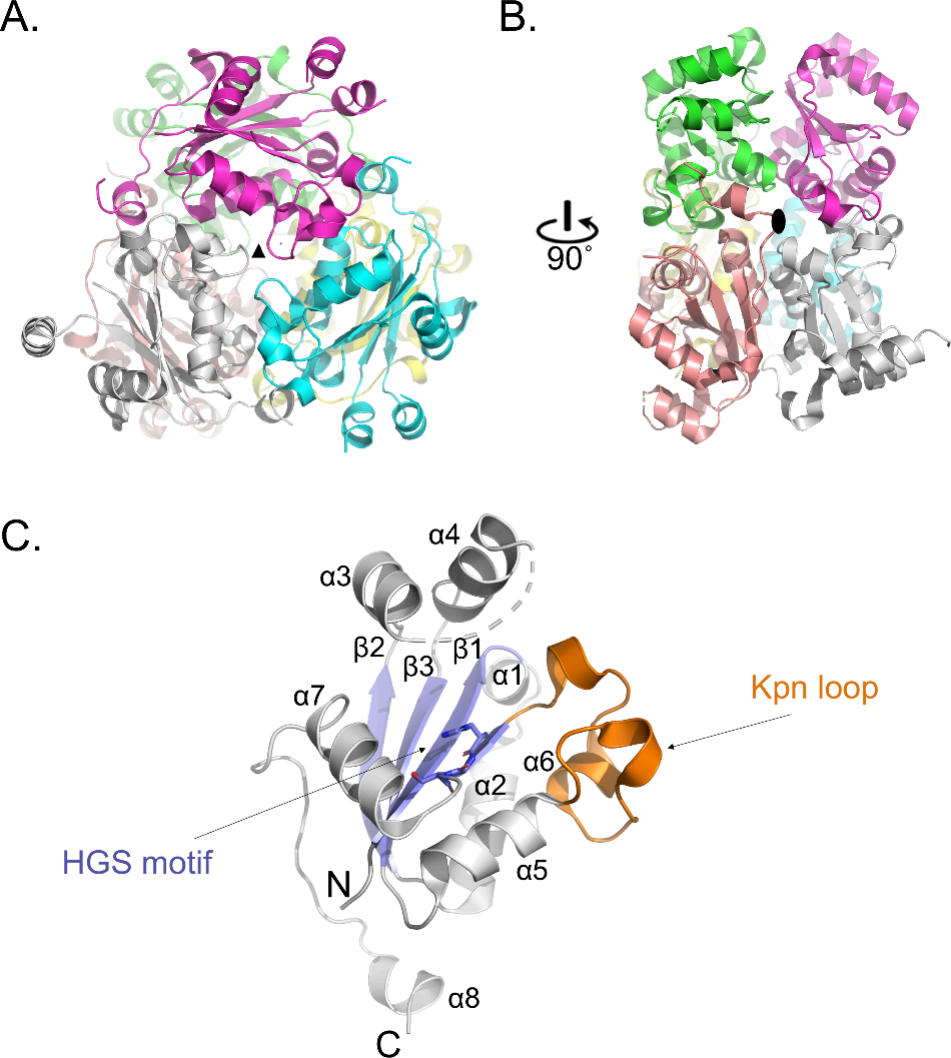
Overall structure of *Tb*NDPK. (a) Cartoon diagram of the hexameric (D3) structure of *Tb*NDPK. Each subunit is represented as a different color (chain A: green, chain B: cyan, chain C: magenta, chain D: yellow, chain E: salmon, chain F: white). The black triangle indicates that the reader is viewing the structure down the 3-fold axis. (b) *Tb*NDPK is rotated 90 °C in which the black oval indicates the reader is viewing down the 2-fold axis. (c) Each monomer is comprised of an a/b sandwich. The helices are gray, and the strands are blue. The Kpn motif is orange and the catalytic HGS motif is shown as blue sticks.

The Kpn-motif of 4F36 is a tightly packed loop that spans residues 91–116 (Figure 2C) and directly precedes the catalytic His117. This region contains two conserved proline residues (Pro95, Pro100) that impose conformational rigidity and limit flexibility of the loop. Interestingly, Val115 adopts unusual main-chain torsion angles which are stabilized by interactions with Asp13 (δO) and Lys11 (amide N). This feature has been seen in other NDPKs.^25,26^ The Kpn-loop not only helps define the shape of the catalytic cleft but also contributes to oligomerization, particularly in stabilizing the trimer interface of the hexamer. This dual role in catalysis and assembly has been observed across multiple NDPK structures and highlights the evolutionary pressure to maintain the loop’s integrity.

### Nucleotide-Binding Mode of *Tb*NDPK

To elucidate the molecular basis of substrate recognition, *T. brucei* NDPK was crystallized in complex with the pyrimidine nucleotides UDP and CDP, as well as the purine nucleotide GDP. In all three complexes, the nucleotide substrates adopt nearly identical orientations within the active site. The diphosphate and ribose moieties form an extensive hydrogen-bonding network (2.7–3.3 Å) with conserved residues Lys11, Arg87, Thr93, Val111, and Asn114 (Figure 3). Arg87 contributes an ionic interaction with the phosphate backbone, whereas Thr93 forms hydrogen-bonds with the β-phosphate. The side chain of Asn114 forms dual hydrogen bonds with the ribose 2′- and 3′-hydroxyls, aided by the backbone carbonyl of Val111 and the ε-amino group of Lys11, which further stabilize the sugar ring (Figure 3). A divalent magnesium ion bridges the α- and β-phosphates, coordinating the ligand and orienting it for catalysis, as reported for other NDPK enzymes.^12^ In addition to these electrostatic and hydrogen-bonding contacts, the nitrogenous base of each NDP participates in aromatic π-stacking with Phe59, positioning the purine and pyrimidine rings coplanar within the pocket. This π-interaction, together with the network of polar contacts, produces a chemically versatile active site that accommodates both base types with minimal energetic discrimination consistent with the substrate promiscuity observed across the NDPK family. An invariant His117, located adjacent to the bound nucleotide, is positioned for phosphoryl transfer, serving as the catalytic nucleophile that forms the transient phospho-histidine intermediate characteristic of this enzyme class.^12^

**Figure 3:**
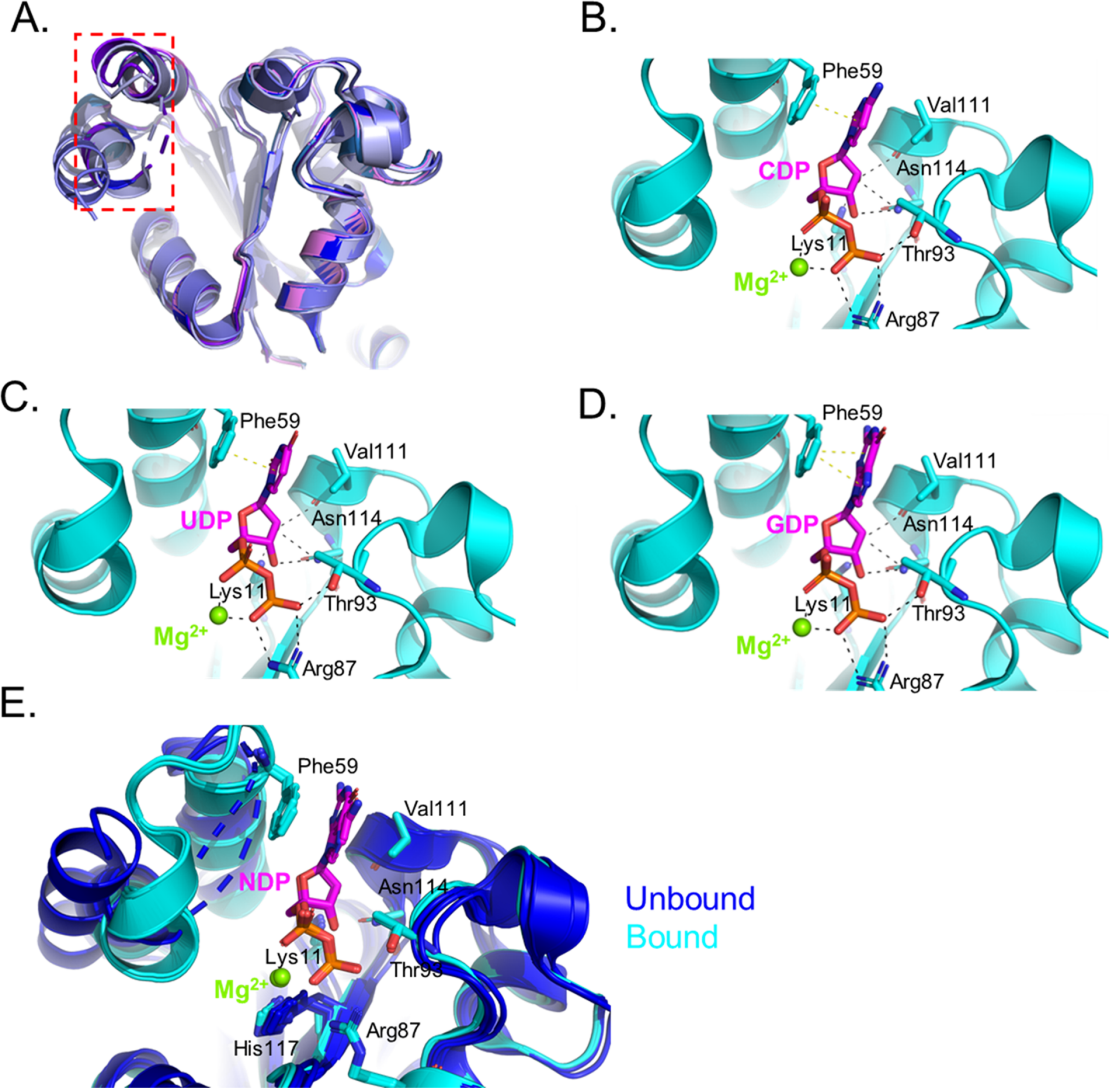
Loop flexibility in *Tb*NDPK hexamer and interactions with substrate. (a) Superposition of monomeric subunits (purple, blue, sky-blue, light blue, and slate) of *Tb*NDPK. The flexible surface loop is highlighted in the red rectangle and shown as dashed lines. Interactions of (b) CDP (c) UDP (d) GDP with *Tb*NDPK (e) superposition of the CDP, UDP, and GDP, with the *apo* structure. The protein is in light blue with the nucleotide substrate shown in magenta. Hydrogen bonding and ionic interactions are shown as black dashed line. Pi-interactions are shown as yellow dashed lines.

In the *apo* structure, continuous electron density was absent for a surface-exposed segment spanning residues 50–69, whose boundaries varied slightly across subunits (A: 51–58; B/C: 51–61; D: 49–61; E: 52–59; F: 57–69). Superposition of the six monomers revealed this segment to form a flexible surface loop that borders the nucleotide-binding pocket and contains the conserved Phe59 (Figure 3A). Upon substrate binding, this region becomes ordered and well resolved in electron density maps, indicating a ligand-induced stabilization (Figure 3E). The conformational change suggests an induced-fit mechanism in which the loop “opens” to admit the nucleotide and “closes” over the active site to secure the substrate through stacking with Phe59. Following phosphate transfer, the loop likely reopens to permit product release. This dynamic loop provides a structural rationale for the enzyme’s broad substrate tolerance. Core catalytic residues recognize the conserved phosphate and ribose groups, while Phe59 ensures proper aromatic stacking with either purine or pyrimidine bases. Our fluorescence-based assays (Figure 1F) also corroborate the structural data, revealing a more closed conformational structure that occurs upon nucleotide binding.

### Enzyme Kinetics and Substrate Specificity of *Tb*NDPK

The kinetic properties of *Tb*NDPK) were investigated using seven nucleotide diphosphate substrates: UDP, CDP, GDP, ADP, dGDP, dCDP, and dADP. Michaelis–Menten kinetics were applied to determine apparent *K*_m_, *V*_max_, *k*_cat_, and catalytic efficiency (*k*_cat_/*K*_m_) for each substrate (Figure 4A and Table S2). *Tb*NDPK displayed the highest substrate preference for UDP, with the lowest *K*_m_ value of 258.9 μM, and a moderate turnover number (2500.0 s^–1^), resulting in the highest catalytic efficiency (Figure 4B). Similarly, *Tb*NDPK also efficiently utilized CDP with a *K*_m_ value of 431.1 μM (Table S2). Interestingly, GDP exhibited a higher turnover rate (*k*_cat_ = 3041.7 s^–1^) than UDP and CDP, but its catalytic efficiency was slightly lower than UDP (Figure 4B). In contrast, ADP was the least preferred ribonucleotide, exhibiting the highest *K*_m_ of 1068 μM, despite a relatively high *k*_cat_ value, resulting in a lower catalytic efficiency. This indicates that while *Tb*NDPK can catalyze ADP turnover efficiently, it does so only at higher substrate concentrations, reflecting reduced substrate preference.

**Figure 4:**
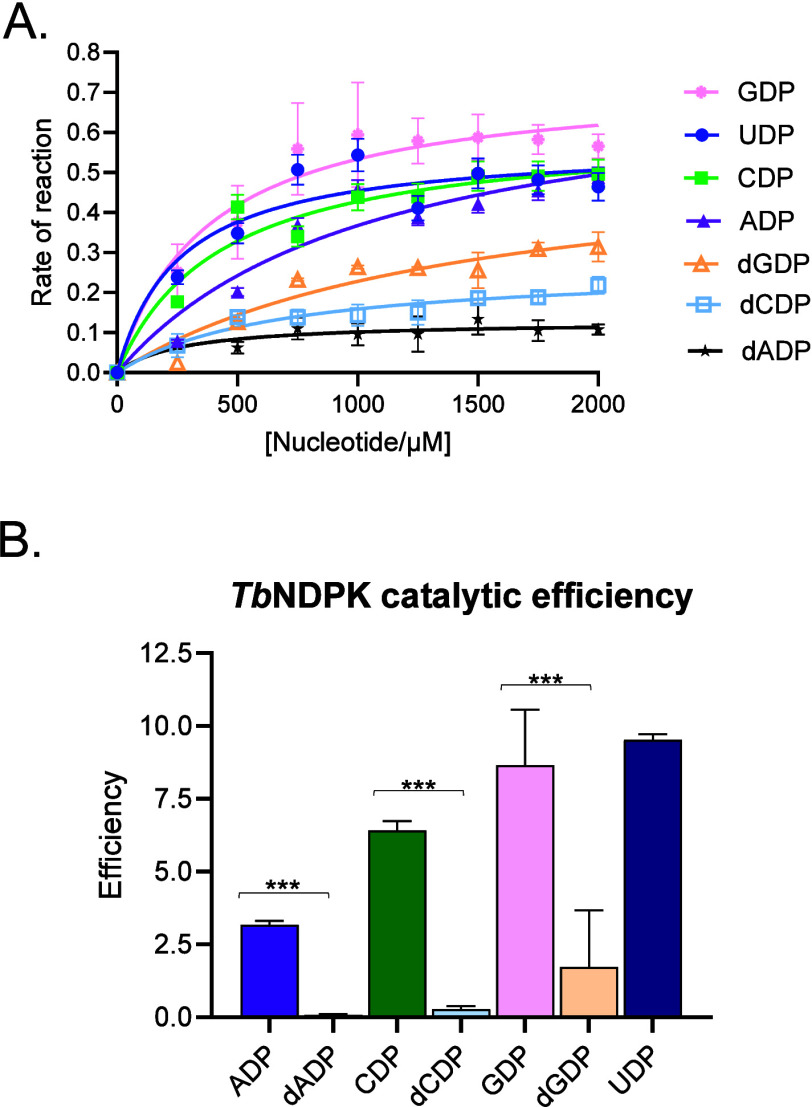
Enzyme kinetics of *Tb*NDPK with various substrates. (A) Michaelis–Menten curves showing the enzyme activity of *Tb*NDPK with seven different substrates: GDP (pink circles), UDP (blue circles), CDP (green squares), dGDP (orange triangles), dCDP (light blue triangles), ADP (purple triangles), and dADP (white squares). (B) Bar graph comparing the catalytic efficiency (*k*_cat_/*K*_m_) of *Tb*NDPK for each substrate. Data represent the mean of three independent replicates. Error bars indicate the standard deviation. Statistical significance is denoted by asterisks (***p < 0.001) after *t* tests with respective NDPs.

Notably, *Tb*NDPK demonstrated the ability to process deoxyribonucleotide diphosphates, although with significantly reduced efficiency (Figure 4B). Both dCDP and dADP showed poor catalytic efficiencies and low *V*_max_ values (Table S2). Nonetheless, dGDP, exhibited higher turnover and catalytic efficiency relative to the other deoxy substrates, though still markedly lower than its ribonucleotide counterpart (Figure 4B). The enzyme kinetics of TbNDPK therefore demonstrate substrate preference for ribonucleotides, particularly UDP and GDP, which are processed with high catalytic efficiency. On the other hand, the enzyme is less efficient with deoxyribonucleotides, suggesting that *Tb*NDPK is evolutionarily optimized for ribose-containing diphosphates, consistent with its physiological role in *T. brucei* nucleotide metabolism. A ranked order of substrate preference based on catalytic efficiency is as follows: UDP > GDP > CDP > ADP > dGDP > dCDP > dADP (Figure 4B).

### Molecular Modeling Studies

Molecular dynamics (MD) simulations were performed to gain deeper insights into the structure–function relationships of *Tb*NDPK. The simulations assessed the structural stability, flexibility, and compactness of the protein, as well as the binding behavior of various nucleotides (ADP, dADP, CDP, dCDP, and UDP) across the six subunits of the hexameric assembly. Each system (the *apo* form and the nucleotide-bound complexes) was subjected to a 1000 ns MD simulation. Simulation quality for all six systems, as indicated by stable potential energy profiles over time, confirmed proper equilibration and reliability of the trajectory data (Figure S3). The Cα root-mean-square deviation (Cα-RMSD) was calculated to evaluate the overall structural stability of *Tb*NDPK during the simulations. The *apo* enzyme reached equilibrium after an initial relaxation phase and thereafter remained within a limited RMSD range over the remainder of the 1000 ns simulation, with an average RMSD of 3.66 Å (Figure 5A). Similarly, all nucleotide-bound complexes remained stable, with average RMSD values of 3.31, 3.53, 3.40, 3.40, and 3.64 Å, for the ADP-, dADP-, CDP-, dCDP-, and UDP-bound *Tb*NDPK, respectively (Figures 5A and S4). These findings further confirm *Tb*NDPK’s inherently robust stability.

**Figure 5:**
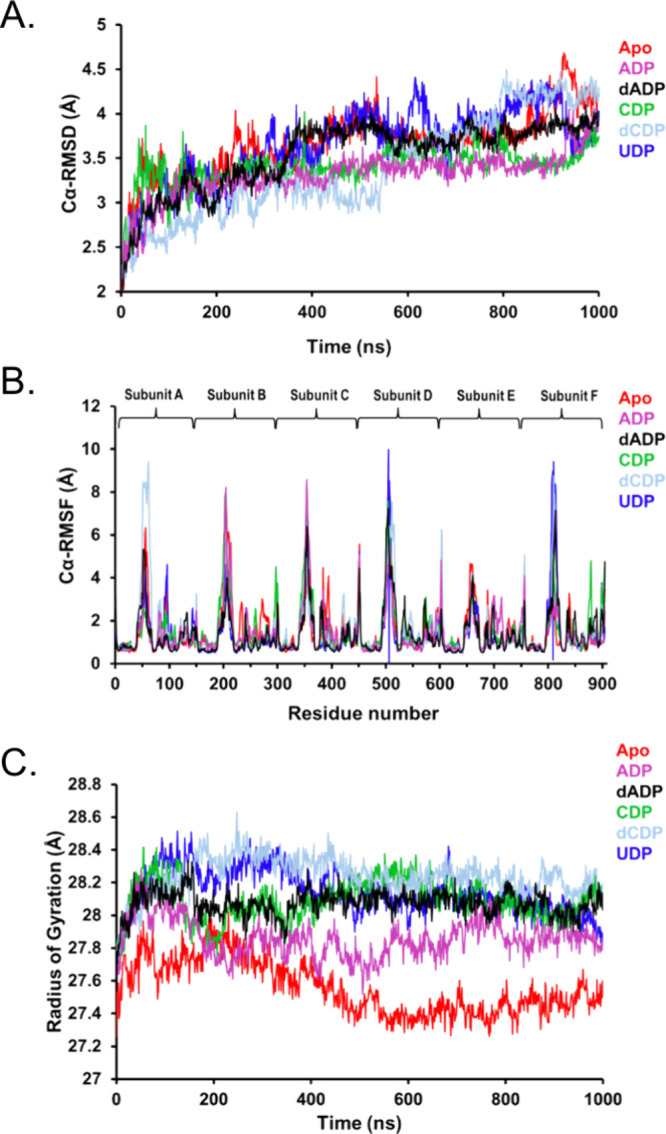
(A) Cα-RMSD of the Cα atoms over time for the six *Tb*NDPK systems, reflecting the average structural deviation from the initial conformation and indicating overall structural stability. (B) Cα-RMSF of the Cα atoms, representing the average positional displacement of each residue within the subunits A–F throughout the simulation. (C) Radius of gyration (*R*_g_) of the six *Tb*NDPK systems, illustrating the overall compactness and conformational changes of the protein, calculated as the root-mean-square distance of all atoms from the center of mass and influenced by ligand binding.

To assess residue-specific fluctuations, the Cα root-mean-square fluctuation (Cα-RMSF) was analyzed for each system (Figure 5B). In the *apo* state, elevated flexibility was observed within specific regions, notably residues 41–67, 195–215, 341–365, 378–383, 449–453, 496–520, 655–671, 797–821, and 905. These regions correspond to dynamic loops within each subunit, as identified in the crystal structure, supporting their intrinsic mobility. Upon nucleotide binding, slight variations in flexibility were observed: the average RMSF values were 1.42 Å (ADP), 1.38 Å (dADP), 1.55 Å (dCDP), and 1.35 Å (UDP), compared to 1.47 Å for the apo structure. Notably, UDP binding resulted in the lowest average RMSF, indicating increased structural rigidity relative to the *apo* form.

The radius of gyration (*R*_g_) was computed to evaluate the global compactness of *Tb*NDPK in different states (Figure 5C). The *apo* structure exhibited the most compact conformation with an average *R*_g_ of 27.56 Å, consistent with *Tb*NDPK’s extensive intersubunit packing observed by crystallography (Figure 2). Upon nucleotide binding, a slight expansion of the protein structure was observed: ADP- and dADP-bound forms exhibited average *R*_g_ values of 27.86 and 28.07 Å, respectively, while CDP- and UDP-bound complexes displayed *R*_g_ values of 28.14 Å. The dCDP complex showed the least compactness with an average *R*_g_ of 28.24 Å. These results suggest that nucleotide binding reduces global compactness, likely contributing to the conformational flexibility observed experimentally. This suggests that *Tb*NDPK maintains high intrinsic stability across conditions but undergoes ligand-specific structural adaptations. To further characterize the binding behavior and dynamics of the nucleotides within the *Tb*NDPK receptor, ligand root-mean-square deviation (ligand-RMSD), molecular surface area (MolSA), solvent-accessible surface area (SASA), and intramolecular hydrogen bonds (intra-HB) were analyzed. Ligand-RMSD provides insight into the internal conformational changes and dynamism of each nucleotide within the receptor pocket.

To further characterize the binding behavior and dynamics of the nucleotides within the *Tb*NDPK receptor, ligand root-mean-square deviation (ligand-RMSD), MolSA, SASA, and intra-HB were analyzed. Ligand-RMSD provides insight into the internal conformational changes and dynamism of the nucleotide within the receptor pocket. The results demonstrate that the five nucleotides exhibit differential dynamic behavior across the six subunits of the enzyme (Figure 6). All five nucleotides displayed enhanced dynamic stability within Subunit A relative to the other subunits (Figure 6). A closer analysis of the conformational disposition of each ligand across the six subunits also affirms the postulate that no ligand will have a similar conformational behavior across the subunits (Figure S5). This finding points to a potential structural–functional independence among the subunits, suggesting that, even within the assembled multimeric complex, individual subunits may preserve a degree of autonomy in their structure–function relationships.

**Figure 6:**
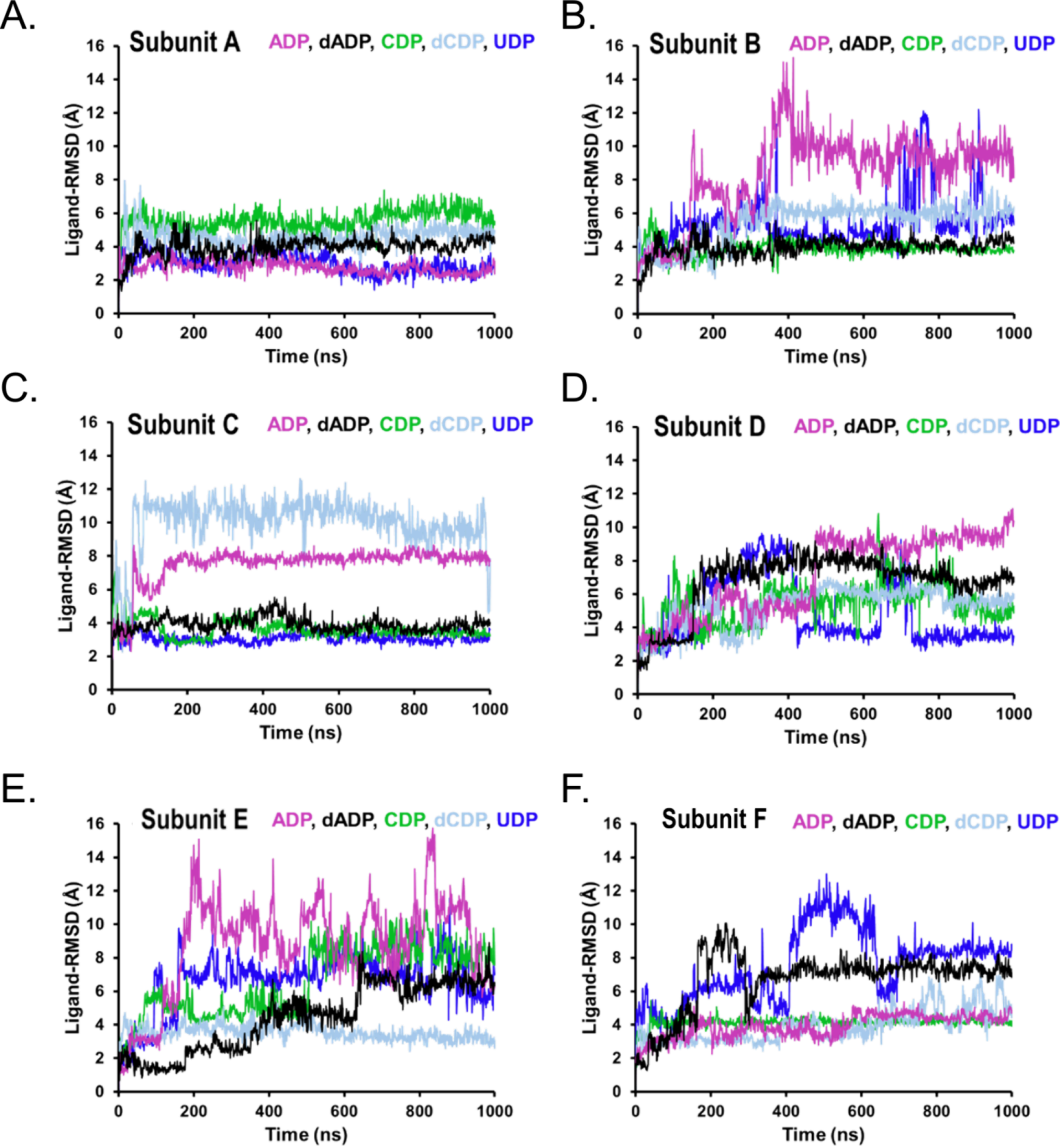
Root-mean-square-deviation of the nucleotides with respect to the receptor sites in each subunit (A–F), which is an index of how much the ligand moves or deviates from its original position over time within the binding pocket.

SASA and MolSA analyses were used to probe complementary aspects of nucleotide behavior within the TbNDPK binding pocket (Figures 7 and 8). SASA reflects the extent of ligand exposure to solvent and therefore provides an estimate of how deeply each nucleotide is buried within the pocket over time. All nucleotides exhibited time-dependent fluctuations in SASA, indicating variable degrees of solvent exposure during the simulations, consistent with the ligand RMSD profiles. ADP and dADP displayed the largest SASA variability, with dADP showing a transient increase in exposure between approximately 350–430 ns across multiple subunits, consistent with a reorientation event rather than ligand dissociation.

**Figure 7:**
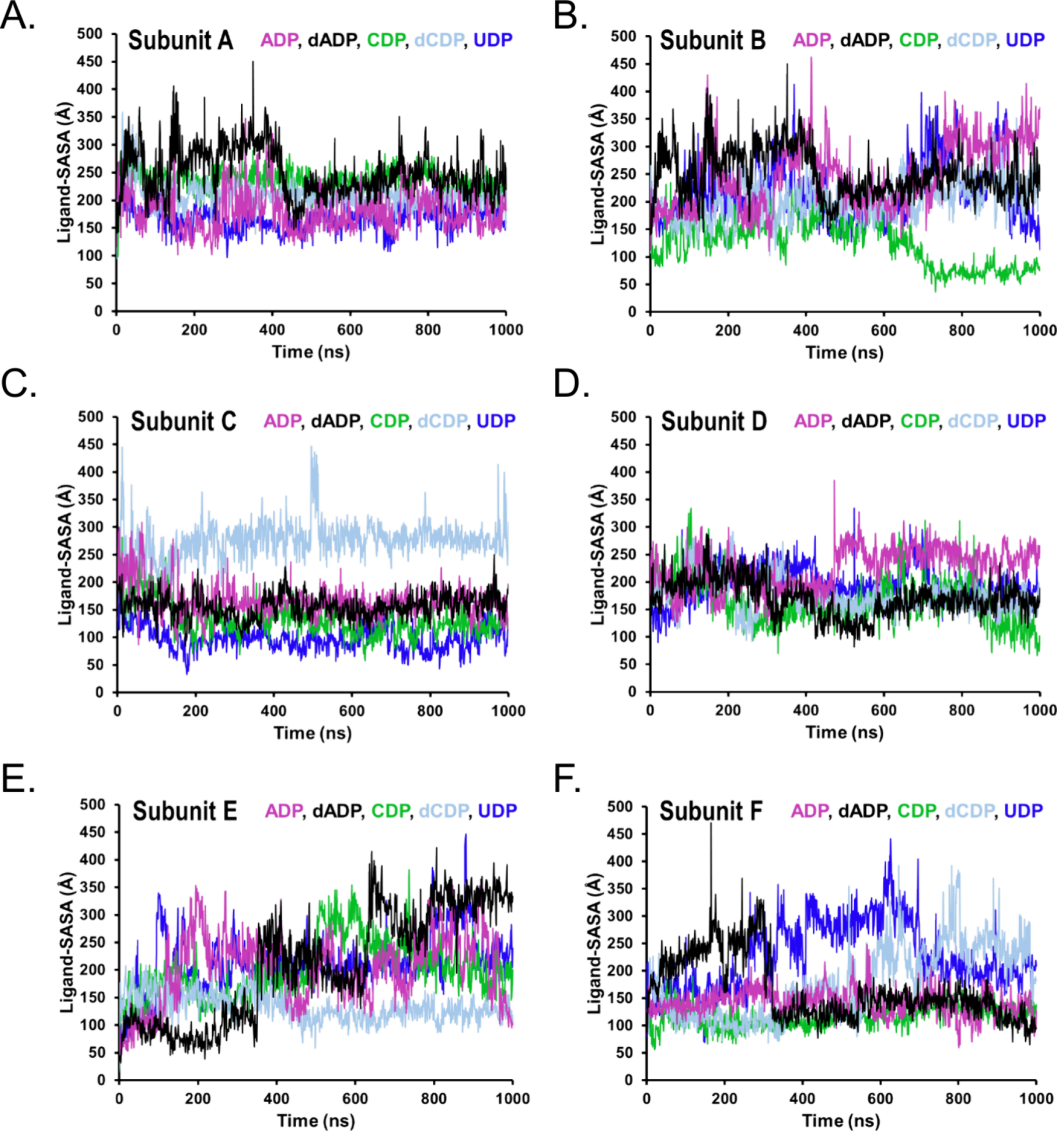
Time evolution of ligand solvent-accessible surface area (SASA) for nucleotides bound to TbNDPK across the six subunits (A–F) of the hexameric complex. SASA reflects the fraction of each nucleotide’s surface that is exposed to solvent during the simulation.

**Figure 8:**
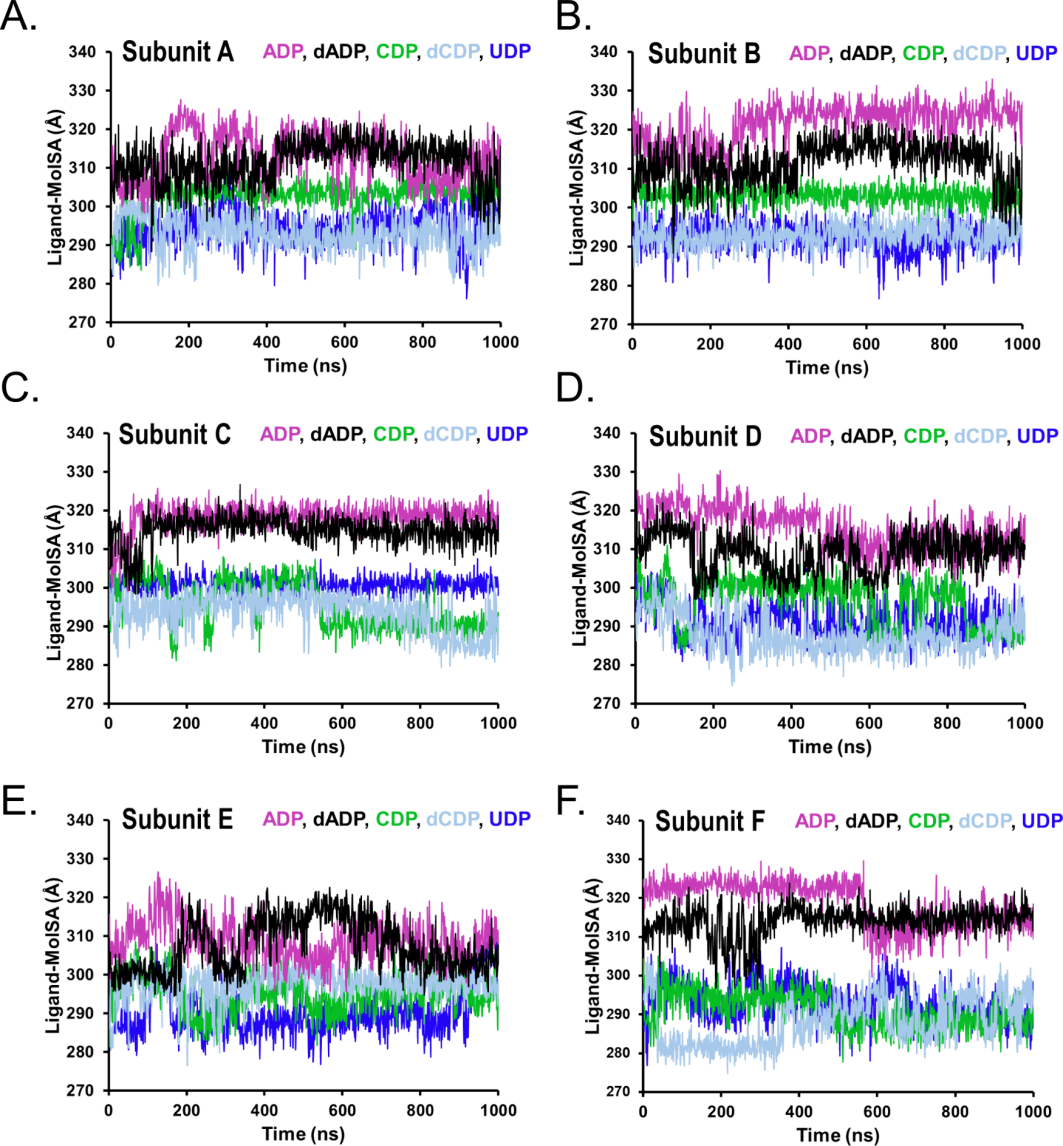
Time evolution of ligand molecular surface area (MolSA) for nucleotides bound to TbNDPK across the six subunits (A–F) of the hexameric complex. MolSA reflects changes in the intrinsic molecular surface of each nucleotide arising from conformational rearrangements and changes in orientation within the binding pocket, independent of solvent accessibility.

In contrast, MolSA reports intrinsic changes in ligand molecular surface arising from conformational rearrangements and changes in orientation, independent of solvent accessibility. MolSA trajectories indicated that CDP, dCDP, and UDP maintained relatively similar intrinsic conformational stability, whereas ADP and dADP exhibited greater surface-area variability, likely reflecting increased flexibility associated with the larger purine base. Together, these analyses indicate that while all nucleotides remain associated with the binding pocket, they differ in both solvent exposure and intrinsic conformational dynamics in a subunit-dependent manner.

Intramolecular hydrogen bond (intra-HB) analysis (Figure S6) offered additional insights into ligand flexibility and internal stabilization. All nucleotides formed a relatively high number of intra-HBs during the simulation, with ADP demonstrating the greatest number of intra-HB interactions and the highest variation across subunits. The variability in intra-HB formation between subunits suggests a heterogeneous binding environment within the hexameric receptor, which is expected given the dynamic nature of protein pockets. Collectively, these analyses reveal that while all nucleotides exhibit some degree of conformational dynamism within the *Tb*NDPK binding pocket, CDP stands out for its initial instability, and ADP and dADP exhibit enhanced flexibility due to structural differences. Overall, the nucleotides were stabilized in the binding pocket via a combination of Hydrophobic interactions, electrostatic interactions due to involvement of the Mg^2+^ in the binding pocket, water bridges and H-bonding, which was the most prominent of the four interactions (Figure S7).

The binding disposition of the nucleotides was determined from their average positions within the binding pocket using trajectory clustering analysis implemented in the Desmond Simulation Engine (Figure S8). The results demonstrate that all five nucleotides occupy the same binding pocket observed in the crystal structure and are stabilized by the identical amino acid residues. Furthermore, the analysis indicates that the Mg^2+^ ion, added to the solvent box during system setup, contributes to nucleotide stabilization (with the exception of ADP), consistent with the interactions reported in the crystal structure.

## Discussion

Nucleoside diphosphate kinases (NDPKs) are multifunctional enzymes that maintain balanced nucleotide pools while participating in diverse cellular processes, including signal transduction, differentiation, and stress adaptation. In *T. brucei*, which lacks a de novo purine biosynthetic pathway, NDPK plays an especially critical role in sustaining nucleotide turnover throughout the parasite’s complex life cycle. The results presented here establish *T. brucei* NDPK (*Tb*NDPK) as a highly stable, catalytically efficient, and structurally specialized enzyme that preferentially acts on ribonucleotide diphosphates, consistent with its role in maintaining RNA-related nucleotide pools.

Biophysical and crystallographic analyses show that *Tb*NDPK maintains its secondary structure at elevated temperatures and displays enhanced resistance to thermal unfolding in the presence of nucleotides (Figure 1). This thermostability likely ensures continuous catalytic activity under the fluctuating thermal and chemical environments encountered during the parasite’s transition between the mammalian host and the tsetse fly vector. The enzyme’s D_3_-symmetric hexameric organization, reinforced by a rigid Kpn-loop, contributes to this stability by promoting intersubunit packing and maintaining the correct catalytic geometry at the trimer interface. Similar oligomeric and structural features have been implicated in the stability of NDPKs from other organisms, including trypanosomatids and *Mycobacterium tuberculosis*.^27^ Notably, TbNDPK lacks the extended N-terminal region present in some eukaryotic homologues, a feature associated with reduced stability, thereby further enhancing its thermal and chemical resistance.^27^ However, given that highly stable NDPKs have been reported in both bacterial and eukaryotic systems,^23^ the absence of this extension is unlikely to be the sole determinant of thermostability.

The structural data reveal that *Tb*NDPK employs the invariant catalytic HGS motif (His117-Gly118-Ser119) supported by an extensive hydrogen-bonding network involving Lys11, Arg87, Thr93, Val111, and Asn114 (Figure 2). Importantly, Asn114 and the backbone carbonyl of Val111 form two hydrogen bonds with the ribose 2′- and 3′-hydroxyl groups of the substrate, conferring a strong stereochemical preference for ribonucleotides. Deoxyribonucleotides, which lack the 2′-OH group, are unable to form these stabilizing interactions, leading to decreased substrate affinity and catalytic efficiency. This structural feature provides a clear mechanistic explanation for the experimentally observed kinetic hierarchy (UDP > GDP > CDP ≫ dGDP > dCDP > dADP; Figure 4B) and highlights how *Tb*NDPK has evolved to favor ribose-based substrates that support rapid RNA synthesis. These interactions rationalize the enzyme’s selective role in ribonucleotide metabolism and its limited participation in DNA precursor turnover. Substrate binding also induces a conformational transition in a surface-exposed loop (residues 50–69) containing Phe59, which forms π-stacking interactions with the nucleobase (Figure 3). In the *apo* structure, this region is flexible and poorly ordered. However, in all nucleotide-bound complexes, it becomes well-defined, supporting an induced-fit mechanism. The loop opens to admit the nucleotide and closes over the catalytic pocket, stabilizing the substrate through π-stacking and polar contacts. The coupling between the rigid Kpn-loop, which maintains catalytic geometry, and this dynamic Phe59 loop, which regulates substrate entry and release, enables *Tb*NDPK to combine high catalytic efficiency with broad substrate tolerance.

Enzyme kinetics reinforce the structural findings from the study (Figure 4 and Table S2). *Tb*NDPK displayed the highest catalytic efficiency for UDP, followed by GDP and CDP, while ADP and all deoxyribonucleotides were processed with markedly reduced efficiency. This substrate ranking aligns with the enzyme’s physiological role in maintaining pyrimidine and purine ribonucleotide pools for RNA synthesis and energy metabolism. The relatively low affinity for ADP compared to UDP and CDP suggests that *T. brucei* primarily relies on pyrimidine substrates, such as UDP, to meet its metabolic demands during growth and differentiation. This is important due to the reliance of trypanosomatids on the de novo pyrimidine biosynthetic pathway. Interestingly, human NDPKs generally exhibit substrate preferences for purine-based NDPs (GDP and ADP).^28^ The significantly lower Km for NDPs versus that for dNDPs could reflect the parasite’s metabolic needs during its lifecycle, where rapid nucleotide turnover might be essential for RNA synthesis and energy metabolism. The lower catalytic efficiencies for deoxy forms suggest that *Tb*NDPK plays a more limited role in DNA metabolism, favoring RNA nucleotide substrates instead. This substrate specificity aligns with the enzyme’s primary role in maintaining nucleotide pools necessary for RNA synthesis and energy metabolism, vital processes for the parasite’s survival and proliferation. However, because different NTPs were utilized as phosphate donors in these assays to avoid substrate competition (ATP for most NDPs and GTP for ADP and dADP), a systematic assessment of the effect of NTP identity on kinase activity will be required in future studies.

To complement empirical data, we performed MD simulations using the experimentally determined hexameric structure of *Tb*NDPK. As an obligate oligomer, *Tb*NDPK requires hexamerization for stability and function, and simulating the intact assembly preserved essential intersubunit interactions (Figure 5). The simulations revealed heterogeneous ligand dynamics across subunits (Figures 5–8), suggesting possible asymmetry or cooperative communication within the hexamer that may fine-tune catalytic efficiency. The simulations revealed ligand-dependent and subunit-specific dynamic behavior. All systems remained globally stable throughout 1000 ns trajectories (average RMSD ≈ 3–4 Å), consistent with the enzyme’s experimental thermostability (Figure 5). The simulations revealed both ligand-dependent and subunit-specific differences within the binding pockets (Figures 5–8), highlighting heterogeneity among symmetry-related subunits within the same oligomeric complex. Such variability likely reflects local differences in pocket flexibility and transient protein–ligand interactions rather than large-scale structural rearrangements.

Analyses of both SASA and MolSA further indicated ligand-dependent differences in solvent exposure and intrinsic conformational flexibility, consistent with subtle nucleotide reorientations within the binding pocket (Figures 7 and 8). These variations are consistent with a dynamic binding environment and do not indicate ligand dissociation. Mg^2+^ ions consistently bridged the α- and β-phosphates of bound nucleotides, stabilizing catalytically competent complexes, except in ADP-bound forms where the ion was absent. This observation agrees with ADP’s rapid product release during catalysis.^29^ Notably, nucleotide dynamics varied between subunits (Figure 6), suggesting potential asymmetry or cooperative effects within the hexamer. These findings indicate that ligand binding at one active site may influence the dynamics of neighboring subunits. Overall, these simulations provide qualitative insight into binding-mode stability, ligand flexibility, and subunit-dependent heterogeneity within the hexameric enzyme. While the observed heterogeneity suggests that individual active sites may sample distinct local conformations, establishing functional coupling or allosteric communication between subunits would require additional targeted simulations and experimental validation.

## Conclusions

Together, these structural, kinetic, and computational findings define *Tb*NDPK as a ribose-selective, thermostable hexameric kinase that integrates rigidity for catalytic precision with flexibility for substrate adaptability. The hydrogen-bond recognition of the 2′-OH group explains the enzyme’s preference for ribonucleotide substrates and provides a molecular basis for its kinetic behavior. This feature also presents a potential avenue for selective inhibitor design. Compounds that disrupt the 2′-OH hydrogen-bonding network or interfere with the Phe59 aromatic stacking could selectively target the parasite enzyme while sparing human homologues. *Tb*NDPK thus emerges as both a model for understanding NDPK substrate discrimination and a promising therapeutic target for antitrypanosomal drug development.

## Materials and Methods

### Protein Production

*Tb*NDPK was cloned, expressed, and purified as previously described.^30–32^ The full-length gene for *Tb*NDPK (Uniprot Q381H3) encoding amino acids 1–153 was PCR-amplified from plasmid using the primers shown below in Table S1. The gene was cloned into the BG1861 expression vector with an N-terminal His tag. The plasmid DNA was transformed into chemically competent *Escherichia coli* BL21­(DE3) Rosetta cells. After testing for expression, 2L of culture was grown using ZYP-5052 autoinduction media^33^ in a LEX Bioreactor (Epiphyte Three) as described previously.^32^

N-terminal hexa-histidine tagged *Tb*NDPK (His-*Tb*NDPK) was purified in the previously described two-step protocol of an immobilized metal (Ni^2+^) affinity chromatography (IMAC) step followed by size-exclusion chromatography (SEC) on an AKTApurifier 10 (GE Healthcare) using automated IMAC and SEC programs.^32^ Briefly, thawed bacterial pellets (25 g) were lysed by sonication in 200 mL lysis buffer (25 mM HEPES pH 7.0, 500 mM NaCl, 5% (v/v) glycerol, 0.5% (w/v) CHAPS, 30 mM imidazole, 10 mM MgCl_2_, 250 mg/mL AEBSF and 0.025% (w/v) sodium azide). After sonication, the crude lysate was treated with 20 mL (25 units/mL) of benzonase and incubated with mixing for 45 min at room temperature. The lysate was clarified by centrifugation at 10,000 rpm for 1 h using a Sorvall centrifuge (Thermo Scientific). The clarified supernatant was then passed over a Ni-NTA HisTrap FF 5 mL column (GE Healthcare), which was pre-equilibrated with wash buffer (25 mM HEPES pH 7.0, 500 mM NaCl, 5% (v/v) glycerol, 30 mM imidazole, 1 mM TCEP, and 0.025% (w/v) sodium azide). The column was washed with 20 column volumes (CV) of wash buffer and eluted with 7 CV of elution buffer (20 mM HEPES pH 7.0, 500 mM NaCl, 5% (v/v) glycerol, 500 mM imidazole). Peak fractions were pooled and concentrated to 5 mL for SEC. The 5 mL protein sample was loaded onto a Superdex 75 26/60 column (GE Biosciences) attached to an ÄKTA Prime-plus FPLC system (GE Biosciences) that was equilibrated with SEC buffer (20 mM HEPES, pH 7.0, 300 mM NaCl, 5% glycerol, and 2 mM DTT). The peak fractions were collected and assessed for purity by SDS-PAGE. His-*Tb*NDPK eluted as a single, symmetrical, monodispersed peak, accounting for >90% of the protein product of molecular mass ∼54 kDa, suggesting purification as a trimer (monomer expected molecular weight 17.6 kDa). The peak fraction was pooled and concentrated to ∼20 mg/mL with an Amicon purification system (Millipore), aliquoted into 110 μL aliquots, flash-frozen in liquid nitrogen, and stored at −80 °C until used.

### Secondary Structure Analyses

The secondary structure and thermal stability of recombinant *Tb*NDPK were analyzed using far-UV circular dichroism (CD) spectroscopy. The experiments were performed using a JASCO J-1500 CD spectrometer (JASCO International, Japan) equipped with a Peltier temperature controller. *Tb*NDPK was analyzed at a final concentration of 0.25 mg/mL in 20 mM PBS, pH 7.5. Where indicated, nucleoside diphosphates (NDPs) were added at a final concentration of 5 mM, and the same concentration was used for all NDPs examined. Far-UV CD spectra were collected using a 0.1 cm path-length quartz cuvette. Spectra were recorded over the wavelength range of 190–260 nm, sampled at 1 nm intervals, with a scan speed of 100 nm min^–1^ and a bandwidth of 1 nm. Each spectrum represents the average of seven accumulations. Baseline correction was performed by subtracting spectra obtained from buffer containing the matched concentration of NDPs but no protein, ensuring that any CD signal contributions from free nucleotides were accounted for. CD data were normalized to protein concentration and expressed as mean residue ellipticity.

Thermal denaturation experiments were conducted to assess secondary structure stability. The unfolding of *Tb*NDPK was monitored at 222 nm while the temperature was increased monotonically from 20 to 90 °C at a rate of 0.5 °C min^–1^. CD signals were recorded at 6 °C intervals, with an equilibration time of 60 s at each temperature prior to data acquisition to ensure thermal equilibration of the sample. The fraction of folded protein at any given temperature was calculated according to eq 1:^34^

$$f_{\mathrm{folded}}=\frac{[\theta {]}_{t}-[\theta {]}_{h}}{[\theta {]}_{l}-[\theta {]}_{h}}$$ 1

where [θ]_*t*_ represents the molar ellipticity at a given temperature, [θ]_*h*_ is the molar ellipticity at the highest temperature (fully unfolded state), and [θ]_*l*_ is the molar ellipticity at the lowest temperature (fully folded state). Melting temperatures (*T*_m_) were determined from the midpoint of the unfolding transition obtained by fitting the temperature-dependent ellipticity data to a two-state unfolding model using GraphPad Prism software.

### Tertiary Structural Conformation Analyses

Next, conformational changes in *Tb*NDPK’s tertiary structure upon nucleotide binding were investigated using intrinsic (tryptophan) fluorescence spectroscopic assays.^35^ Recombinant *Tb*NDPK was used at a final concentration of 0.5 mg/mL and incubated in the absence or presence of saturating concentrations of nucleotides (5 mM NDPs or dNDPs) for 15 min at room temperature prior to data acquisition. All samples were prepared in assay buffer (25 mM HEPES-KOH, pH 7.5, 100 mM KCl, 10 mM MgOAc). Fluorescence measurements were performed in a quartz cuvette (1 cm path length) using a JASCO FP-6300 spectrofluorometer. Tryptophan fluorescence emission spectra were collected between 305 and 500 nm following excitation at 295 nm, using a scan speed of 500 nm min^–1^. To minimize photobleaching effects, each sample was exposed to excitation light only once. Data acquisition was carried out using JASCO Spectra Manager software, and spectral analysis was performed using the same software package. To control for potential contributions of free nucleotides to the observed fluorescence signal, buffer-only controls containing the corresponding concentrations of nucleotides (5 mM) but no protein were measured under identical conditions and subtracted from the protein-containing spectra. In addition, *Tb*NDPK spectra acquired in the absence of nucleotides were used as internal controls to assess nucleotide-induced spectral changes. All experiments were performed in triplicate using independently prepared protein samples, and representative spectra or averaged emission maxima were used for comparative analysis. Nucleotide-induced conformational changes were assessed by monitoring shifts in emission maxima and changes in fluorescence intensity.

### X-ray Crystallography

Three crystal forms of *Tb*NDPK are reported, and all crystals grew by vapor diffusion in sitting drops using Tray 101d6, 96 well plates under conditions described in Table 1. Data collection and processing parameters are summarized in Table 2.

**Table 1:** Crystallization

**crystal form**	**4F36 Apo**	**4F4A,** UDP complex	**4FKX,** CDP complex	**4FKY,** GDP complex
temperature (K)	291	291	291	289
protein concentration (mg/mL)	20.2 mg/mL	35.4 mg/mL	35.4 mg/mL	20.2 mg/mL
buffer composition of protein solution	20 mM HEPES, pH 7.0, 300 mM NaCl, 5% glycerol and 2 mM DTT	20 mM HEPES, pH 7.0, 300 mM NaCl, 5% glycerol and 2 mM DTT	20 mM HEPES, pH 7.0, 300 mM NaCl, 5% glycerol and 2 mM DTT	20 mM HEPES, pH 7.0, 300 mM NaCl, 5% glycerol and 2 mM DTT
composition of reservoir solution	20% PEG3350, 200 mM sodium isothiocyanate	20% PEG3350, 200 mM magnesium formate, 2 mM UDP, 10 mM magnesium chloride	40% MPD, 5% PEG8000, 0.1 M sodium cacodylate, pH 6.5, 2 mM CDP, 10 mM magnesium chloride	40% MPD, 5% PEG8000, 0.1 M sodium cacodylate, pH 6.5, 2 mM CDP, 10 mM magnesium chloride
volume and ratio of drop	0.4 μL, 1:1	0.4 μL, 1:1	0.4 μL, 1:1	0.4 μL, 1:1
volume of reservoir (μL)	80 μL	80 μL	80 μL	80 μL
composition of cryoprotectant solution	directly from crystallization buffer	directly from crystallization buffer	directly from crystallization buffer	directly from crystallization buffer

**Table 2:** Data Collection and Processing^a^

**data set**	**4F36 Apo**	**4F4A,** UDP complex	**4FKX,** CDP complex	**4FKY,** GDP complex
source	SSRL beamline BL7-1	RIGAKU FR-E+ SUPERBRIGHT	RIGAKU FR-E+ SUPERBRIGHT	RIGAKU FR-E+ SUPERBRIGHT
wavelength	1.0332	1.5418	1.5418	1.5418
detector	ADSC quantum 315r CCD	DECTRIS PILATUS 6M-F	RIGAKU SATURN 944+	RIGAKU SATURN 944+
data collection scaling software	XSCALE	XSCALE	XSCALE	XSCALE
data reduction software	XDS	XDS	XDS	XDS
space group	*P*2_1_2_1_2_1_	*C*222_1_	*C*222_1_	*C*222_1_
*a*, *b*, *c* (Å)	52.46, 123.67, 145.36	71.83, 121.51, 113.17	71.63, 121.37, 112.87	71.68, 121.49, 112.83
α, β, γ (°)	90, 90, 90	90, 90, 90	90, 90, 90	90, 90, 90
resolution range (Å)	50–2.3 (2.36–2.30)	41.74–2.10 (2.15–2.10)	50–1.70 (1.74–1.70)	50–1.95 (2.00–1.95)
completeness (%)	98.8 (91.3)	98.1 (91.9)	98.1 (90.2)	98.5 (85.8)
redundancy	4.3 (3.1)	6.7 (6.5)	8.5 (8.0)	6.4
⟨*I*/σ(*I*)⟩	12.97 (2.0)	23.16 (7.5)	32.77 (3.24)	23.6
*R*_r.i.m._	0.088 (3.78)	0.08 (0.257)	0.079 (0.438)	0.066

a Values for the outer shell are given in parentheses.

The three structures were determined by molecular replacement with Phaser from the CCP4 suite of programs.^36–39^ Structure quality was checked with MolProbity.^40^ Data-reduction and refinement statistics are shown in Table 3. Coordinates and structure factors have been deposited with the Worldwide PDB (www.PDB.com).

**Table 3:** Structure Solution and Refinement^a^

**structure**	**4F36 Apo**	**4F4A,** UDP complex	**4FKX,** CDP complex	**4FKY,** GDP complex
search model	3R9l	4F36	4F36	4F36
phasing software	PHASER	PHASER	PHASER	PHASER
refinement software	REFMAC	REFMAC	REFMAC	REFMAC
resolution range (Å)	50.0–2.30 (2.36–2.30)	41.74–2.10 (2.54 −2.10)	41.33–1.70 (1.74–1.70)	50–1.95 (2–1.95)
completeness (%)	98.9 (98.9)	98.1 (98.1)	98.1 (98.1)	98.1 (85.8)
no. of reflections, working set	42483 (2541)	28728 (1784)	53338 (3286)	35706 (2023)
no. of reflections, test set	2144 (131)	1444 (101)	2628 (140)	1777 (102)
final *R*_cryst_	0.198 (0.262)	0.158 (0.167)	0.152 (0.239)	0.148 (0.168)
final *R*_free_	0.235 (0.323)	0.195 (0.230)	0.182 (0.243)	0.188 (0.196)
No. of Non-H Atoms				
protein	6311	3499	3526	3515
ion	0	3	3	3
ligand	18	80	80	92
solvent	236	416	516	499
total	6600	3993	4128	4109
R.M.S. Deviations				
bonds (Å)	0.014	0.013	0.011	0.012
angles (°)	1.41	1.15	1.525	1.523
Average *B* Factors (Å^2^)				
protein	41.7	27.9	13.8	12.5
ion	47.0	25.6	17.3	15.1
ligand		23.6	19.3	31.0
water	35.9	25.3	24.4	22.3
Ramachandran Plot				
most favored (%)	98	98	98	98
allowed (%)	1	1	1	1
outlier (%)	1	1	1	1

a Values for the outer shell are given in parentheses.

### Kinase Activity Assays

*Tb*NDPK kinase activity assays were carried out by incubating 0.2 μM recombinant *Tb*NDPK for 1 h at 25 °C in activity buffer (10 mM HEPES-KOH, pH 7.5; 100 mM KCl; 2 mM MgCl_2_; 0.5 mM DTT) with NDP/dNDP substrates at concentrations ranging from 0 to 2 mM. Five mM ATP was utilized as phosphate donor, except for dADP and ADP where 5 mM GTP was added. Boiled *Tb*NDPK samples served as nonenzymatic controls to account for spontaneous ATP hydrolysis. For ADP/dADP measurements, the Transcreener Kinase Assay with an Alexa Fluor-conjugated GDP antibody was used, enabling real-time detection of ADP formation through competition with a fluorescent tracer. Similarly, Alexa Fluor-conjugated ADP antibodies were utilized for UDP, CDP, GDP and their dNDP forms. Following excitation at 580 nm, fluorescence was measured at 620 nm emission. Each assay was performed in triplicate using independent protein batches. Kinetic parameters (*K*_m_ and *V*_max_) were then derived by fitting Michaelis–Menten curves with GraphPad Prism 6.05.

### Molecular Docking

Molecular docking studies were performed by Schrödinger (maestro) software to investigate binding scores and interactions formed between compounds and protein crystal structures. Coordinates of the crystal structures of 4F36 (apo), 4F4A (UDP- bound), 4FKX (CDP bound), and 4FKY (GTP bound) were retrieved from the protein data bank (PDB) and prepared for docking by adding partial charge, potential energy, minimized the energy and removed water molecules. The PubChem Web site (https://pubchem.ncbi.nlm.nih.gov/) was used to screen compound libraries with a similar chemical formula as the UDP compound (https://pubchem.ncbi.nlm.nih.gov/) and prepared for interaction using Ligprep. The receptor generation (glide) was used to select the cocrystal ligand in each protein for binding site formation. Thereafter, ligand docking was used; the generated receptor grid zip file and ligands in the project table were selected. The complexes generated with the highest binding score were selected and viewed in ligand interaction for the formed interactions between protein and ligand (Hoda et al.).

### Molecular Simulation

#### Preparation of *Tb*NDPK-Nucleotide Complexes

The coordinates for the unbound hexameric *Tb*NDPK protein were retrieved from the RCSB Protein Data Bank (PDB ID: 4F36) and imported into the Maestro v13.6 molecular modeling suite for preparation using the Protein Preparation Wizard. The structure underwent standard preprocessing steps, including assignment of bond orders, addition of hydrogen atoms, creation of zero-order bonds for metals and disulfide linkages, and removal of water molecules within 5 Å of heteroatoms (nonstandard residues such as ions or solvent molecules). To optimize the hydrogen bonding network, the orientation of water molecules was sampled using the PROPKA algorithm at pH 7.0. Water molecules forming fewer than three hydrogen bonds with nonwater atoms were deleted. The structure was then energy minimized using the OPLS4 force field, with restraints applied to heavy atoms. The minimization was terminated once the root-mean-square deviation (RMSD) of the heavy atoms reached a convergence threshold of 0.3 Å. The stereochemical integrity of side chains was carefully examined to ensure no significant perturbations occurred during preparation. The final minimized *Tb*NDPK structure was saved in Maestro (.mae) format for subsequent ligand docking studies.

UDP was prepared for docking. Briefly, the 3D structure of UDP was extracted from the *Tb*NDPK-UDP complex (PDB ID: 4F4A) and submitted to the LigPrep module in Maestro v13.6. Ligand preparation included energy minimization using the OPLS4 force field. The algorithm was configured to generate all relevant protonation and ionization states of UDP at pH 7.0 ± 0.2, with p*K*_a_ values predicted using the Epik module. Additionally, the ligands were desalted, and up to three tautomeric states were generated per ligand at pH 7.0 ± 0.2. For molecules with multiple chiral centers, specified stereochemistry was retained where needed, while undefined centers were allowed to vary in order to generate chemically plausible, low-energy conformation of UDP. The resulting UDP structure was saved as an individual Maestro (.mae) file for subsequent induced-fit docking (IFD) studies.

IFD, as implemented in Schrödinger Maestro v13.6, was employed to predict the binding of UDP to each of the six subunits of the prepared *Tb*NDPK protein. The binding site was defined based on the orientation of UDP in the crystal structure (PDB ID: 4F4A). Docking was carried out using an implicit solvent model with the OPLS4 force field. During IFD, ring conformational sampling was applied with a 2.5 kcal/mol energy barrier, and a nonplanarity penalty was enforced on amide bonds to ensure chemically realistic ligand geometries. The scaling factors for both the receptor and the ligand were set to 0.5, and a maximum of 10 poses per ligand was allowed. Residues within 5.0 Å of the docked ligand were further refined using the Prime Refinement algorithm. The resulting protein–ligand complexes were ranked based on Prime energy scores. Receptor conformations within 30.0 kcal/mol of the lowest energy structure were selected for a final round of Glide docking and scoring. In this second docking step, UDP was redocked into each refined low-energy receptor structure using the default Glide XP (extra precision) settings.

To generate the remaining four *Tb*NDPK–nucleotide complexes, the bound UDP in the original *Tb*NDPK structure was systematically modified to ADP, dADP, CDP, and dCDP using the Build Tool in Maestro v13.6. Specifically, UDP in each subunit was first modified to CDP, and the resulting structure was saved as a Maestro (.mae) file. CDP was then further modified to dCDP by adjusting the sugar moiety. In parallel, UDP was modified to ADP by altering the nitrogenous base. This ADP–*Tb*NDPK complex was subsequently converted to dADP. All generated complexes were then preprocessed, optimized, and energy-minimized at pH 7.0 ± 0.2 using the OPLS4 force field, prior to molecular dynamics simulations.

### Molecular Dynamics Simulation

MD simulations were conducted using the Desmond engine in a high-performance computing system accelerated by an Nvidia RTX-4080 GPU. Briefly, the docked and modified nucleotide complexes, along with the unbound (*apo*) protein structure, were transferred to a Linux (Ubuntu) system for Desmond simulation. The simulation workflow comprised two main phases: system preparation (including solvation and ionization) and production run. During the preparation phase, the System Builder module in Desmond was used to solvate each system with the TIP3P explicit water model and apply the OPLS_2005 force field. Each complex was placed in an orthorhombic simulation box with a 10 Å buffer from the outermost atom to the box edge (box angles α = β = γ = 90°). The box volume was minimized, and counterions were added to neutralize the system. To simulate physiological ionic condition, MgCl_2_ was added to the solvent environment. Following solvation and ionization, each system underwent a six-stage calculation and equilibration protocol, as follows: stage 1: task analysis and precalculation; stage 2: simulation using the Brownian Dynamics with the *NVT* ensemble, *T* = 10 K, small timesteps, and restraints on solute heavy atoms for 100 ps; stage 3: simulation using *NVT* ensemble, *T* = 10 K, small timesteps, and restraints on solute heavy atoms for 12 ps; stage 4: simulation using the *NPT* ensemble, *T* = 10 K, and restraints on solute heavy atoms for 12 ps; stage 5: simulation using the *NPT* ensemble and restraints on solute heavy atoms for 12 ps; stage 6: simulation using the *NPT* ensemble with no restraints for 24 ps. This was followed by the MD production run, consisting of a 1000 ns MD simulation under *NPT* ensemble conditions, representing the primary data generation phase for downstream analysis. This was followed by the MD production run, consisting of a 1000 ns MD simulation under *NPT* ensemble conditions, representing the primary data generation phase for downstream analysis.

### Post-Dynamic Trajectory Analysis

Postsimulation analyses were conducted to evaluate the structural dynamics and stability of the *Tb*NDPK-nucleotide complexes in comparison with the unbound (*apo*) protein. Trajectory files generated from the MD simulations were subjected to a series of quantitative assessments using tools available in the Desmond simulation package and Maestro v13.6. Key parameters analyzed included the Cα root-mean-square deviation (Cα-RMSD) to monitor global structural stability, and the Cα root-mean-square fluctuation (Cα-RMSF) to evaluate residue-level flexibility over the course of the simulation. The radius of gyration (RoG) was computed to determine the compactness of the protein structure. To assess ligand dynamics, the nucleotide RMSD with respect to the receptor was calculated as a function of time, reflecting the stability and positional retention of the nucleotide within the binding pocket. Furthermore, the molecular surface area (MolSA) and solvent-accessible surface area (SASA) of each nucleotide were computed throughout the trajectory to quantify changes in ligand exposure and interaction with the solvent environment. In addition to these parameters, a ligand, protein contact summary was generated to identify key residues involved in binding and to evaluate the persistence and nature of these interactions during the simulation. These analyses provided comprehensive insights into the dynamic behavior and binding stability of each complex under physiological simulation conditions.

## Supplementary Material











## Notes

Crystallographic data for 4F4A (CIF)
Crystallographic data for 4F36 (CIF)
Crystallographic data for 4FKX (CIF)
Crystallographic data for 4FKY (CIF)
Additional experimental methods; protein purification and characterization data; supplementary circular dichroism and fluorescence spectroscopy analyses; extended multiple sequence and structural alignments; and additional molecular dynamics analyses (PDF)
