# Supplementary material for: Structural and Functional Analyses of Trypanosoma brucei Nucleoside Diphosphate Kinase
Source: ACS Omega. 2026 Mar 9;11(11):17663–77. doi: 10.1021/acsomega.5c11614 (PMC13019236; doi:10.1021/acsomega.5c11614)
Supplement: Supplementary file 5 [file ao5c11614_si_005.pdf]

Supplementary information for Structural and Functional Analyses of *Trypanosoma brucei* Nucleoside Diphosphate Kinase

Patricia Makori[1], Michael P. Boeckman[2], Heidi S. David[2], Finley Payne[3], Markenya Gatling[1], Colby Greer[1], Dylan Hayes[1], Alexandra Jefferson[1], Micaela Maxwell[1], Christian Smith[1], Jamilah Watson[1], London Williams[1], Jazmin Barkley[1], Caitlyn Pepper[1], Tawanda Zininga[4], Sandhya Subramanian[5,6], Ariel Abramov[6], Anna. S. Gardberg[7], Thomas E. Edwards[6,7], Bart L. Staker[5,6], Lance J. Stewart[7], Peter. J Myler[5,6], Oluwatoyin Asojo[6,8], Olamide Jeje[9], Sylvia Fanucchi[9], Ikechukwu Achilonu[9], Craig L. Smith[2*] and Graham Chakafana[1*]

[1]Department of Chemistry and Biochemistry, Hampton University, Virginia, USA

[2]Department of Biology, Washington University in St. Louis, MO, USA

[3]The Governor's School, Hampton, Virginia, USA

[4]Stellenbosch University, Department of Biochemistry, Stellenbosch, South Africa

[5]Center for Global Infectious Disease Research, Seattle Children's Research Institute, 307 Westlake Avenue North Suite 500, Seattle, USA

[6]Seattle Structural Genomics Center for Infectious Disease (SSGCID), Seattle, Washington, USA

[7]Beryllium Discovery Corp, Bainbridge Island, WA 98110, USA

[8]Dartmouth Cancer Center & Department of Biochemistry and Cell Biology, Dartmouth Giesel School of Medicine, Lebanon, New Hampshire, 03756 USA

[9]Protein Structure-Function Research Laboratory, School of Molecular and Cell Biology, University of the Witwatersrand, Braamfontein 2050, Johannesburg South Africa

*Corresponding author

graham.chakafana@hamptonu.edu

csmith22@wustl.edu

Table S1. Macromolecule production information

Source organism	*Trypanosoma brucei TREU927*
Forward primer	5'CTCACCACCACCACCACCATATGCCCAGCGAGCGGACCTTC3'
Reverse primer	5'ATCCTATCTTACTCACTTATGCCCTTTCATAAATTTGCTTGACG3'
Expression vector	BG1861
Expression host	*Escherichia coli* BL21(DE3) Rosetta
Complete amino acid sequence of the construct produced.	MAHHHHHHMPSERTFIAVKPDGVQRNLVGEIIKRFENKGYKLVGLKLLQPTEEQAKQHYIDLASKPFYSGLVSYFSSGPIVGMVWEGLGVVKGGRVLLGATNPADSLPGTIRGDFAVDVGRNVCHGSDSVESAKREIAFWFKAEELVSWTSHSVKQIYERA

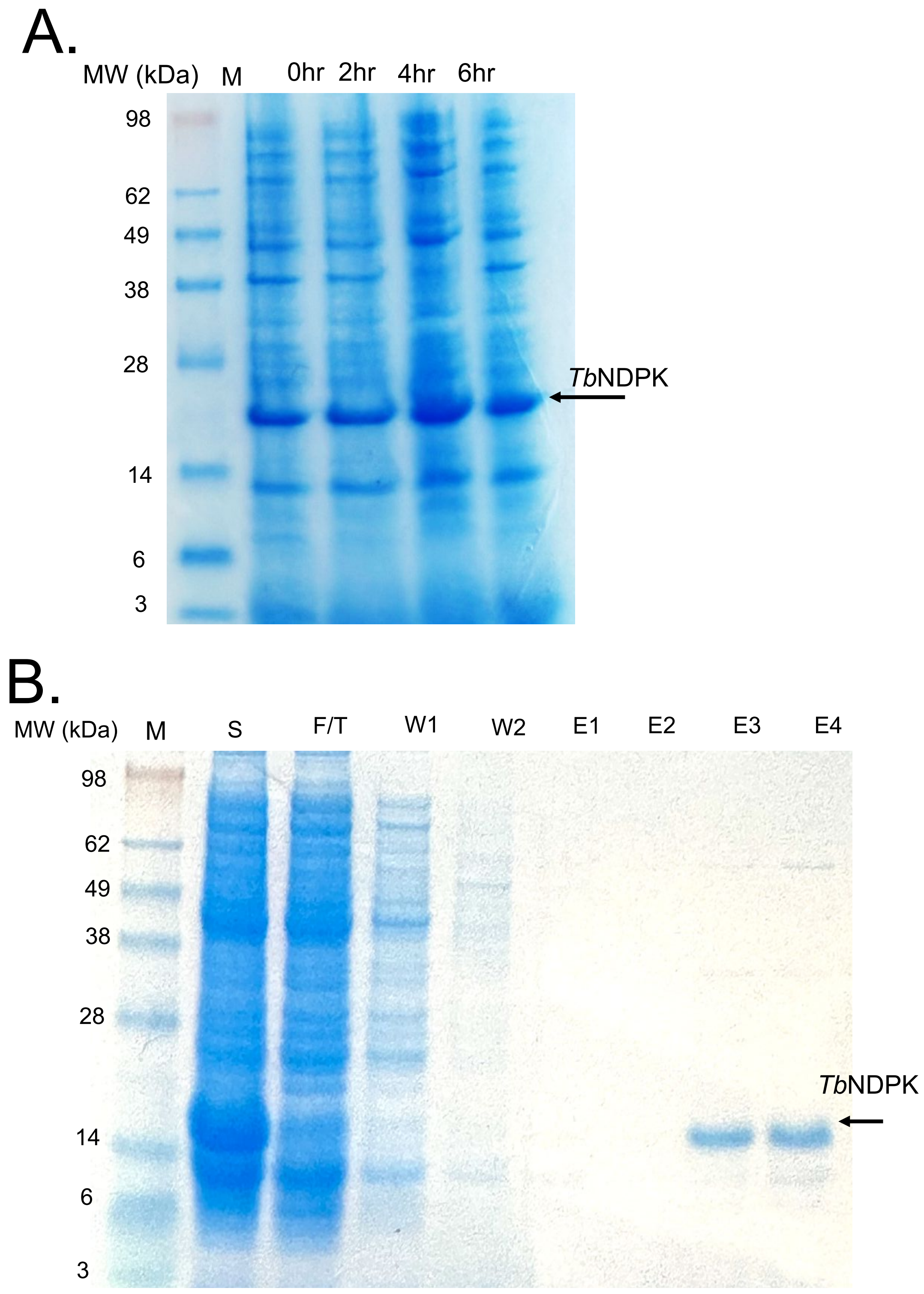

Figure S1. (A) SDS-PAGE analysis for the expression of *Tb*NDPK. Lanes include the molecular weight marker (M) with weights in kDa. Samples were collected at different time intervals post-induction: 0 hr (pre-induction), 2 hr, 4 hr, and 6 hr post-induction. (B) Purification of *Tb*NDPK using affinity chromatography. The gel shows the molecular weight marker (M) and various fractions, supernatant (S), flowthrough (F/T), washes (W), and elutions (E1 - 4).

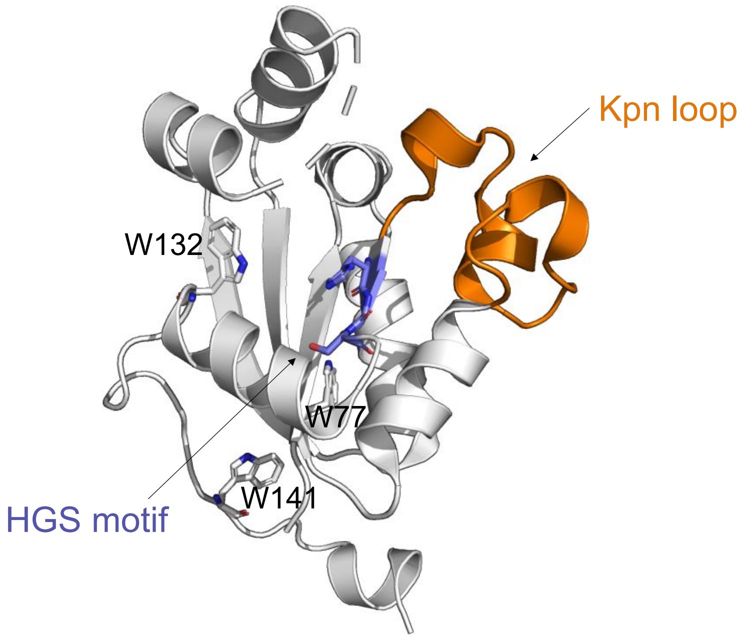

Figure S2. Distribution of tryptophan residues in TbNDPK.

Table S2. Enzyme activity of *Tb*NDPK

**Substrate**	**Km (µmol^{-1}) +/- SD**	**Vmax (µmol/min/mg) +/- SD**	**Kcat (s^{-1})**	**Catalytic efficiency (M^{-1}S^{-1})**
**UDP**	258.9 +/-25	0.60 +/-0.09	2500.0	9.66×10^6
**CDP**	431.1 +/-37	0.64 +/-0.13	2666.6	6.19×10^6
**dCDP**	272.5 +/-14	0.13 +/-0.03	54.17	1.99×10^5
**ADP**	1068+/-41	0.79 +/-0.2	3291.7	3.08×10^6
**dADP**	684.7+/-36	0.13 +/-0.04	57.41	8.38×10^4
**GDP**	377.6+/-65	0.73+/-0.03	3041.7	8.05×10^6
**dGDP**	266.8+/-10	0.12+/-0.07	500.0	7.30×10^5

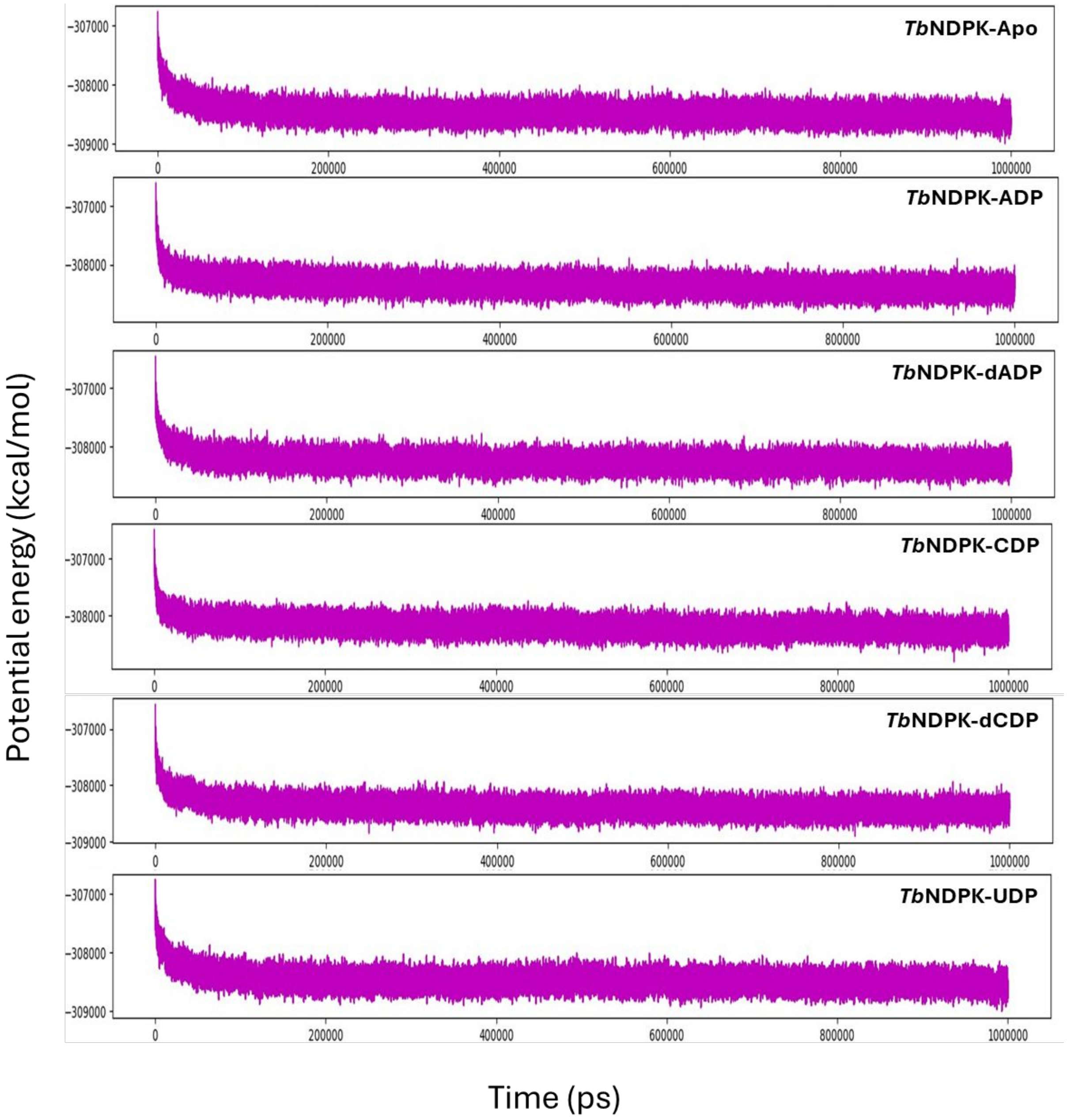

Figure S3: Plots of the potential energy as a function of time, indicating that all six systems were properly equilibrated during the simulation.

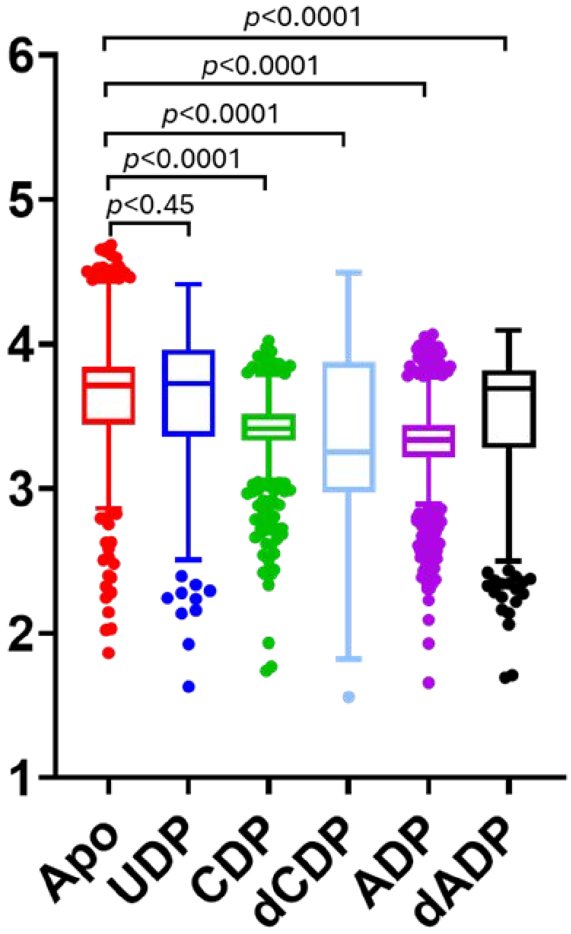

Figure S4: Tukey box-and-whisker plots representing the distribution of Cα-RMSD values for each system over the entire simulation time.

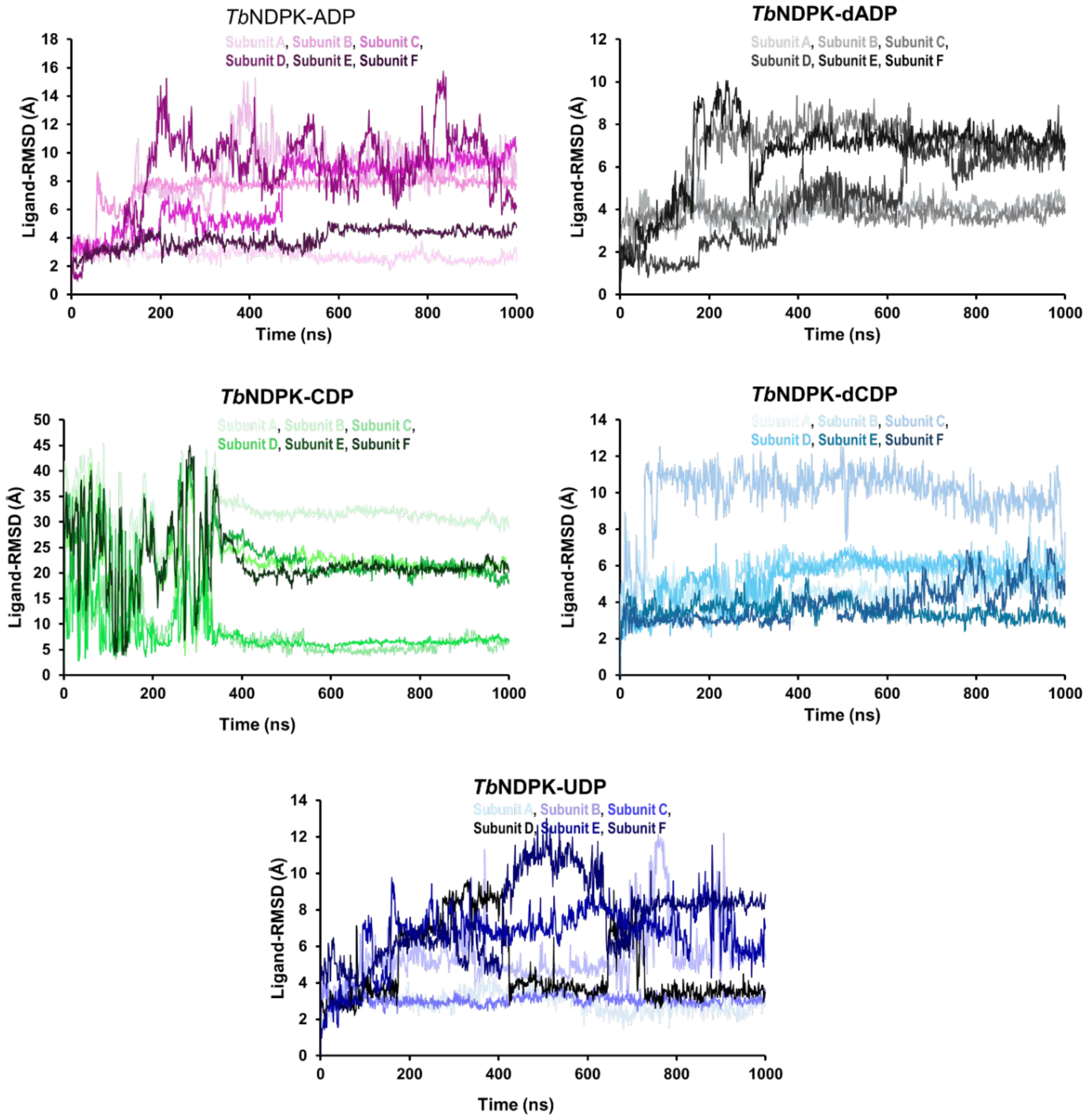

Figure S5: Solvent-accessible surface area (SASA) of the ligand over the 1000-ns simulation time, illustrating the variation in the dynamics of each nucleotide across the six subunits of *Tb*NDPK.

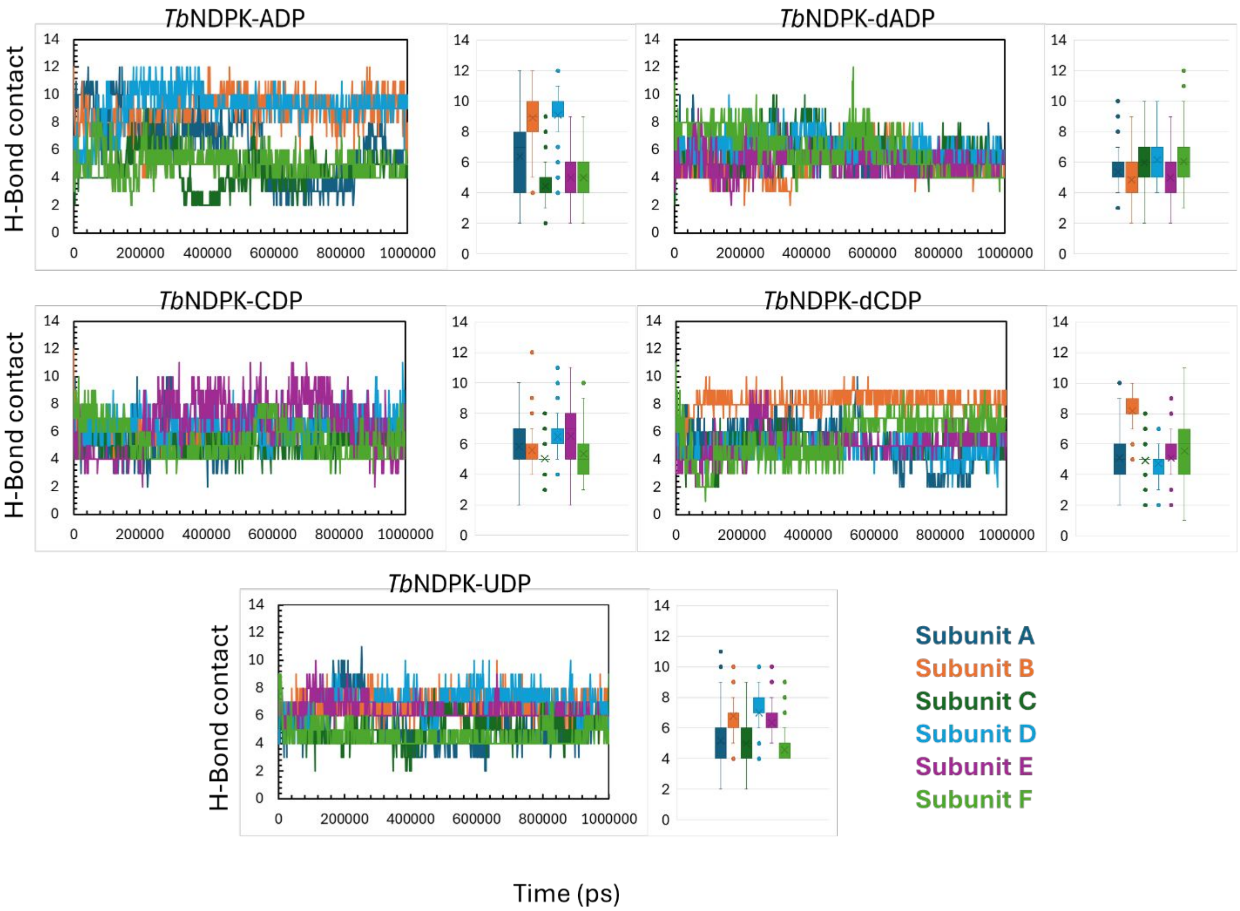

Figure S6: Hydrogen bond analysis of the ligand within the binding pocket across the simulation trajectory. Variations in the number of hydrogen bond contacts reflect the dynamic nature of ligand-protein interactions.

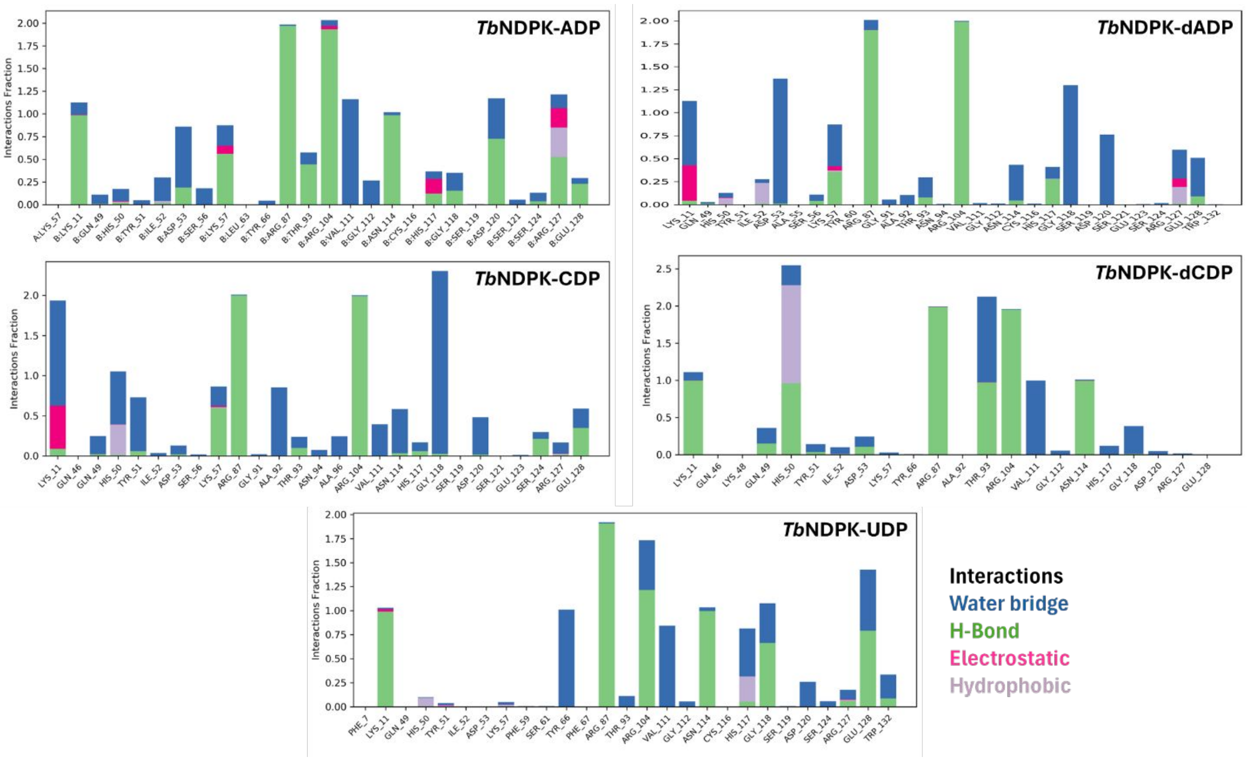

Figure S7: Stacked bar plots of the specific interactions stabilizing each nucleotide in subunit A (as the reference subunit) across the five *Tb*NDPK-nucleotide complexes.

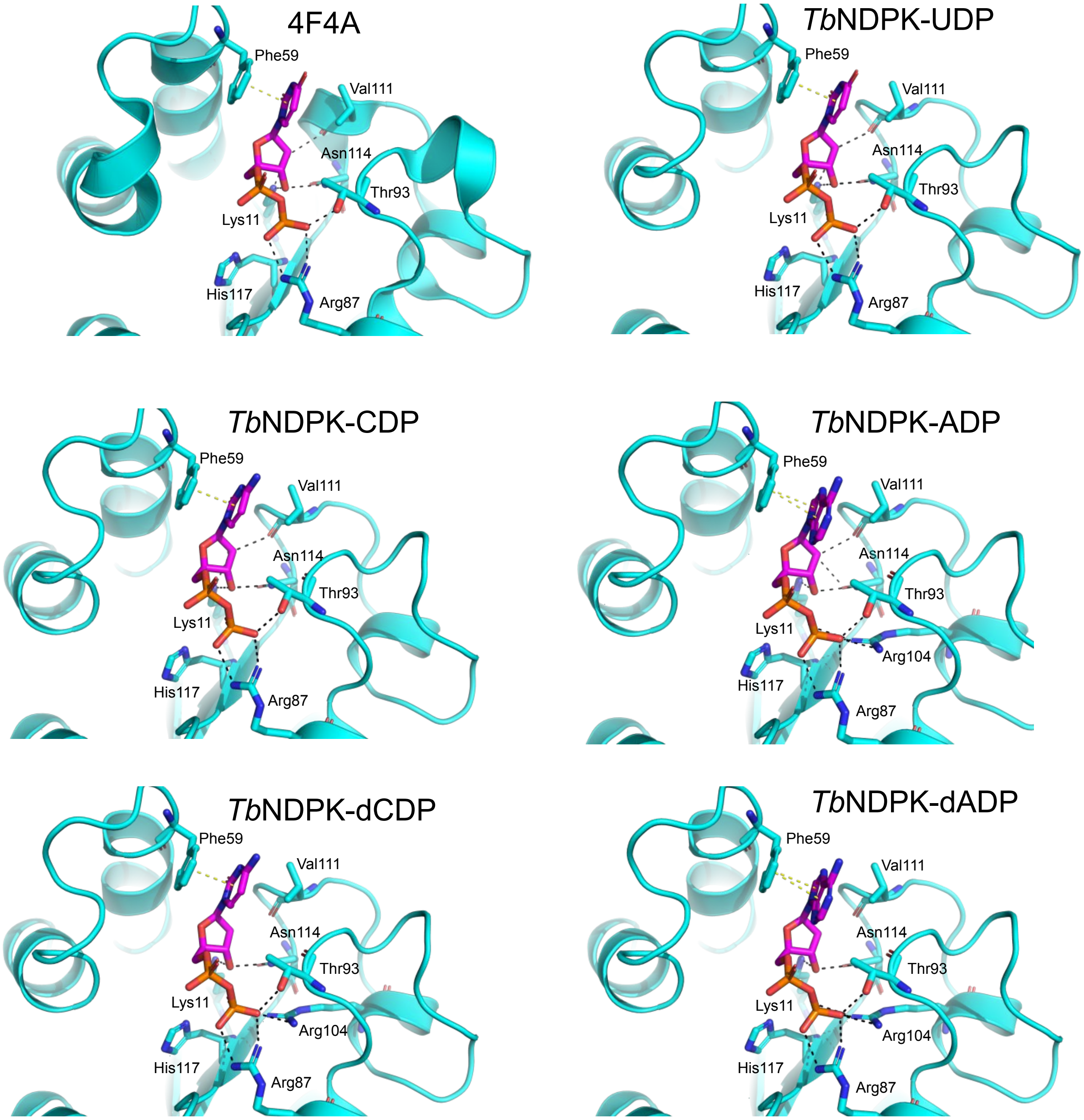

Figure S8: The average positions of nucleotides within the binding pockets of five *Tb*NDPK-nucleotide complexes, compared to the reference crystal structure (PDB ID: 4F4A). The figure demonstrates that all five nucleotides occupy binding sites similar to those observed in the experimentally determined structure, highlighting consistency in binding pocket location.
